# A Modular and Programmable Cas13d Platform for RNA Single Nucleotide Variant Detection

**DOI:** 10.1002/advs.202523680

**Published:** 2026-05-04

**Authors:** Zeyu Wang, Jiahao Li, Zhuying Yue, Beixuan He, Wenhua Zhang, Zhen Fang, Qing Xia, Yingbin Liu, Yanjing Li

**Affiliations:** ^1^ State Key Laboratory of Systems Medicine For Cancer Shanghai Cancer Institute Renji Hospital Affiliated to Shanghai Jiao Tong University School of Medicine Shanghai China; ^2^ Shanghai Key Laboratory of Cancer System Regulation and Clinical Translation (CSRCT‐SHANGHAI) Jiading District Central Hospital Shanghai China; ^3^ Department of Oncology Renji Hospital Affiliated to Shanghai Jiao Tong University School of Medicine Shanghai China; ^4^ Department of Biliary Pancreatic Surgery Renji Hospital Affiliated to Shanghai Jiao Tong University School of Medicine Shanghai China; ^5^ Department of General Surgery, Jiading Branch Renji Hospital Shanghai Jiao Tong University School of Medicine Shanghai China; ^6^ Department of Hepatobiliary and Pancreatic Surgery Affiliated Hospital of Zunyi Medical University Guizhou China

**Keywords:** Cas13d RNA diagnostics, CRISPR platform, precision oncology, programmable, single nucleotide variant detection

## Abstract

CRISPR‐based nucleic acid diagnostics have shown broad potential, yet reliable single‐nucleotide variant (SNV) discrimination remains limited by flanking sequence requirements that constrain targetability, and an inherent specificity‐sensitivity trade‐off where mismatch designs used to suppress wild type recognition often penalize enzymatic activity. Here we develop a scenario‐guided Cas13d framework that supports pre‐defined operating modes tailored to distinct analytical goals. Leveraging the minimal protospacer flanking site constraints of Cas13d, we first map mismatch‐sensitive windows to derive rule‐based crRNA designs that improve allelic discrimination. We then restore assay performance through structure‐guided engineering of a miniaturized Cas13d scaffold by internally inserting auxiliary RNA binding domains (RBDs). Systematic benchmarking across representative oncology hotspots delineates two practical regimes comprising an ultra‐sensitive, amplification‐free mode in which a dual‐RBD variant paired with optimized mismatched crRNAs achieves ∼0.6% variant allele fraction (VAF) detection, and a robust amplified mode incorporating optional loop‐mediated isothermal amplification coupling that favors simpler architectures to balance performance and background across broader low‐VAF ranges. In an evaluation of 45 clinical tumor RNA specimens spanning pancreatic, cholangiocarcinoma, and colorectal cancers, the assay correctly classified mutation status with full concordance for KRAS G12D, IDH1 R132C and BRAF V600E, with a subset of positive cases corroborated by orthogonal RT‐ddPCR. A prospective IDH1 R132C clinical‐matrix spike‐in further supported sub‐1% detection without pre‐amplification. Collectively, this work establishes a configurable Cas13d toolkit and a rule‐guided strategy for deploying CRISPR‐based RNA SNV diagnostics with application‐specific performance objectives.

## Graphical abstract

A scenario‐guided Cas13d platform enables configurable single nucleotide variant ribonucleic acid diagnostics through rule‐based guide design and ribonucleic acid binding domain engineering, supporting amplification‐free ultrasensitive detection and amplification‐coupled robust detection for accurate mutation classification in clinical tumor specimens.

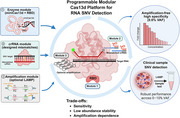

## 1 Introduction

Single nucleotide variants (SNVs) within cancer driver genes are among the most recurrent and clinically actionable alterations in oncology. Hotspot substitutions in genes such as *KRAS*, *BRAF*, and *IDH1* can refine diagnosis, guide targeted therapy, and inform prognosis [1, 2, 3, 4, 5]. However, detecting these SNVs in routine practice is challenged by specimen heterogeneity, variable tumor cellularity, and low mutant allele fractions. This creates a practical need for assays that provide reliable single‐nucleotide discrimination while remaining compatible with clinical workflows. Current clinical SNV testing is dominated by quantitative PCR, droplet digital PCR, and next generation sequencing [6, 7, 8, 9]. Although these methods achieve high analytical performance, they rely on specialized instrumentation, multi‐step workflows, and centralized infrastructure. The associated turnaround times and costs limit repeated measurements and large‐scale screening, especially for longitudinal monitoring or in resource‐limited settings. These constraints have motivated interest in simplified molecular readouts that can be executed with fewer steps while retaining single nucleotide resolution.

CRISPR‐based diagnostics help address this need by coupling programmable base pairing to enzymatic signal generation, translating sequence recognition into a measurable output [10, 11, 12, 13]. For SNV discrimination, nuclease choice is critical because sequence constraints and mismatch‐sensitive architectures dictate targetable loci and optimal variant positioning within a guide (Figure S1A,B). Cas12a operates on DNA and is constrained by a strict protospacer adjacent motif (PAM) requirement [14, 15, 16]. It also relies on a short, mismatch‐intolerant PAM‐proximal seed region, which can restrict guide design when targeting fixed clinical variants (Table 1). RNA‐targeting Cas13 enzymes provide an alternative by operating on transcripts and activating a HEPN RNase upon target binding, enabling collateral reporter cleavage as a built‐in amplification mechanism [12, 13, 17, 18, 19]. However, Cas13 orthologs differ substantially in flanking sequence preferences and intrinsic mismatch sensitivity profiles. Cas13a orthologs used in established diagnostic workflows often exhibit protospacer flanking site (PFS) preferences and mismatch sensitivity that peaks in the mid‐spacer region [20, 21]. When both PFS constraints and positional rules are applied, a substantial fraction of SNV loci cannot be aligned into an optimal discrimination window. In contrast, RfxCas13d is largely PFS‐independent and shows reproducible position‐dependent mismatch penalties [22, 23, 24]. This expands the guide‐design space and increases the fraction of clinically relevant SNVs that can be positioned within a sensitive window. In an oncologic context, applying hotspot SNVs to practical guide‐design rules illustrates that Cas13d increases site targetability compared with PAM‐ or PFS‐limited effectors, motivating Cas13d as a suitable scaffold for a broadly reusable SNV detection module (Figure S1C,D) [11, 12, 13, 18, 21, 25]. In addition, by directly interrogating target transcripts, this RNA‐targeting platform can provide complementary information beyond DNA genotyping in selected contexts [6, 7, 9]. While DNA genotyping remains the clinical standard, RNA readouts can confirm expression of a mutant allele and capture transcript‐level events such as gene fusions and splice‐altering consequences. Accordingly, RNA‐based testing serves as an orthogonal tool that can support clinical interpretation when transcript‐level confirmation is important.

**TABLE 1:** Comparison of SNV detection characteristics between engineered Cas13d platform and existing Cas systems.

Feature	Cas13a (RNA)	Cas12a (DNA)	Cas13d (RNA)
**Sequence Constraint**	**PFS‐restricted**: ortholog‐dependent PFS preference (e.g., LshCas13a requires a 3′ non‐G PFS)	PAM‐restricted: strict PAM requirement for target recognition (e.g., canonical TTTV/TTTN PAM for many Cas12a)	PFS‐independent: no strict PFS requirement reported for Cas13d under standard guide designs
**Mismatch Sensitivity**	Position‐dependent: Most sensitive in mid‐spacer; Ends more tolerant	Seed‐dependent: PAM‐proximal seed most intolerant; Distal more tolerated	Position‐dependent: Distal impacts binding; Proximal impacts activation
**SNV Site Targetability**	Moderate: Constrained by PFS + Mismatch window	Low: Constrained by PAM + Seed rules	High: No PFS allows precise SNV Alignment into the sensitive window
**Protein Size**	1150–1390 aa	1228–1307 aa	930–970 aa engineered mini RfxCas13d: 682 aa
**Catalytic** **Behavior**	HEPN RNase: Moderate on‐target RNA cleavage + High‐turnover collateral ssRNA	RuvC DNase: High‐efficiency on‐target dsDNA cleavage + Low‐turnover collateral ssDNA	HEPN RNase: Strong on‐target RNA cleavage + Robust collateral ssRNA

**Table footnote. SNV site targetability** is defined as the ability to position the variant nucleotide within a mismatch‐sensitive window given PAM/PFS constraints.

Despite the favorable design space of Cas13d, reliable single nucleotide discrimination at low variant allele fraction (VAF) remains difficult. Many Cas13 workflows rely on mismatch‐engineered guides to suppress wild type recognition [12, 13, 25]. However, mismatches that improve specificity often reduce overall activity by weakening binding, impairing activation, or lowering turnover [18, 26]. This creates a specificity‐sensitivity trade‐off that is especially restrictive in oncology, where actionable variants can occur at low VAF and background RNA complexity is high. The central technical challenge is therefore to sharpen allelic discrimination without losing the catalytic output needed for detection.

Protein engineering provides a complementary axis because Cas13 activity depends on both base pairing and protein‐mediated target engagement followed by conformational activation of the HEPN nuclease [27, 28, 29]. Strengthening target engagement or stabilizing productive target‐bound states could offset the activity losses imposed by mismatch‐engineered guides, preserving the base‐pairing rules that encode specificity. To explore this concept, we leveraged miniCas13d, a compact RfxCas13d derivative that retains RNA‐guided activity [30]. This miniaturized scaffold is advantageous for modular fusion designs, providing additional flexibility for incorporating auxiliary domains.

We focused on auxiliary RNA binding domains (RBDs) as modular additions [31, 32]. We selected a panel spanning three canonical RNA binding folds, RRM, KH, and C3H1‐type zinc finger domains, because they are compact, autonomously folding modules that engage short stretches of single‐stranded RNA through distinct binding chemistries [33, 34, 35]. Specifically, RRMs typically use a β‐sheet surface to contact a few nucleotides. KH domains bind short motifs through a conserved loop and adjacent helices. C3H1‐type zinc fingers can recognize AU‐rich RNA features in a context‐dependent manner. Importantly, many members of these families exhibit limited sequence preferences rather than strict motif requirements, making them suitable as general tethering elements across diverse target sequences.

In this study, we develop a scenario‐guided Cas13d framework that treats guide design, protein engineering, and optional isothermal amplification as tunable modules that can be configured for a given objective. First, we map mismatch‐sensitive windows in Cas13d to derive rule‐based crRNA designs that improve single nucleotide discrimination across representative oncology hotspots. Second, we restore and elevate assay output via the structure‐guided integration of auxiliary RBDs into miniCas13d, targeting sterically permissive sites identified through modeling of the Cas13d ternary complex. Third, we consider LAMP as an optional module that can improve sensitivity in specific contexts but can also raise guide‐dependent background, requiring configuration‐specific tuning [16, 36]. Together, this modular approach provides a configurable and practical strategy for deploying Cas13d‐based RNA SNV diagnostics across diverse variants and workflow constraints.

## 2 Results

### 2.1 Design and Validation of SNV Targeting crRNAs

**FIGURE 1:**
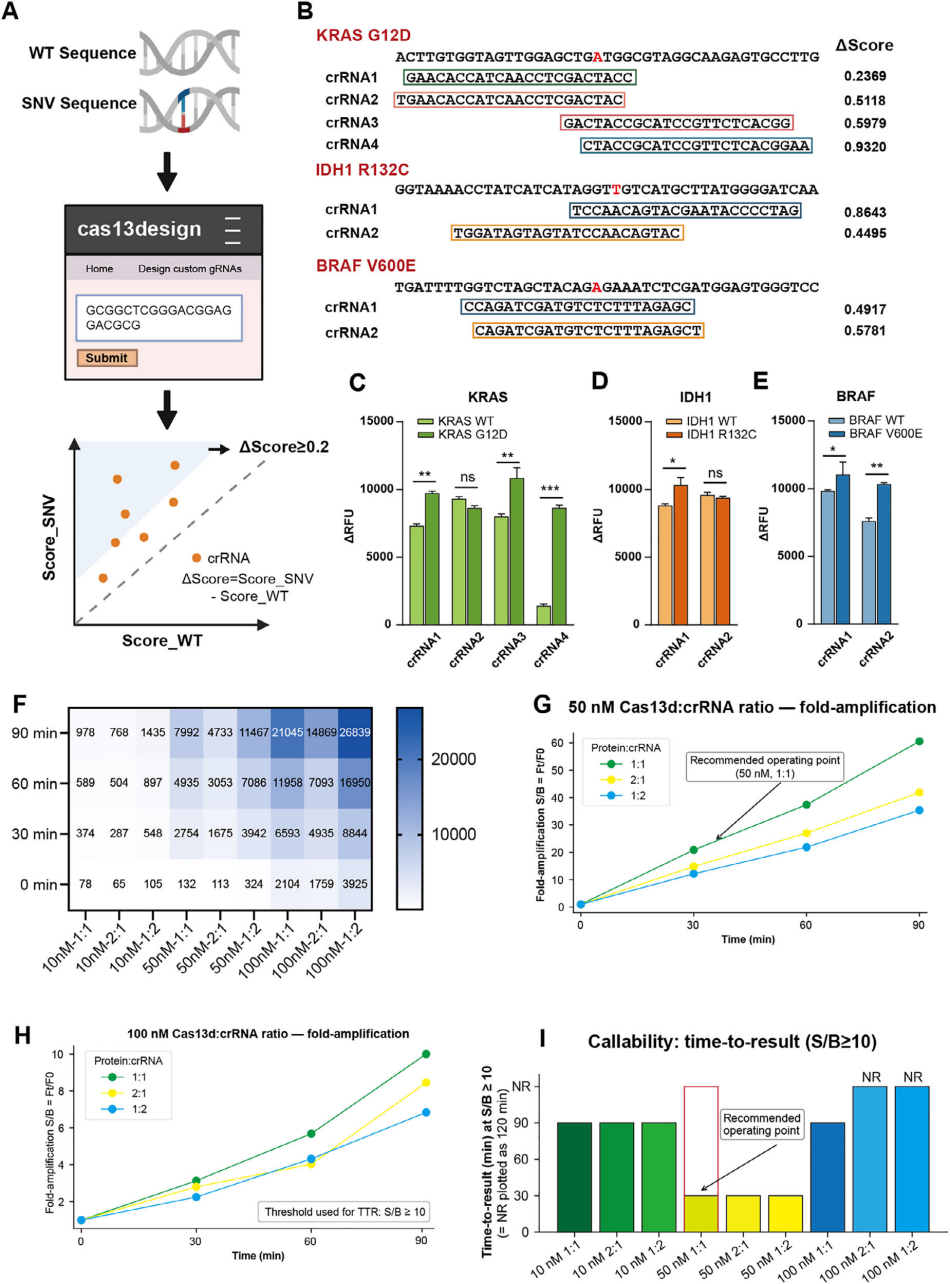
Design and evaluation of SNV specific crRNAs using the Cas13d Design platform. (A) A schematic illustrating SNV specific crRNA design using the publicly available Cas13design tool (http://cas13design.nygenome.org) [38], which predicts Cas13d cleavage activity based on sequence features. Activity scores were computed for both wild type and mutant targets, and the difference (ΔScore = Score_SNV—Score_WT) was used to estimate SNV selectivity. crRNAs with ΔScore ≥ 0.2 were prioritized for testing. (B) Candidate crRNAs targeting *KRAS* G12D, *IDH1* R132C, and *BRAF* V600E. For each target, 2–4 crRNAs with varying ΔScores were selected. SNV sites are highlighted in red; targeted regions are boxed. ΔScore values reflect the predicted discriminatory power between SNV and WT sequences. crRNA spacer sequences are shown in the 3’ to 5’ orientation to illustrate base pairing with the target sequence. (C–E) Fluorescence cleavage assays for each crRNA against wild type and SNV RNA targets. ΔRFU was calculated as RFU at 90 min minus RFU at 15 min. Data are presented as mean ± SD (n = 3). Statistical significance was assessed using independent samples *t*‐tests. * *p* < 0.05; ** *p* < 0.01; *** *p* < 0.001; ns, not significant. Among the tested crRNAs, *KRAS* crRNA4 (C), *IDH1* crRNA1 (D), and *BRAF* crRNA2 (E) showed the largest signal differences between WT and SNV targets, consistent with their predicted ΔScores. (F) Heatmap of fluorescence signals across a Cas13d and crRNA titration matrix. Reactions were assembled with Cas13d at 10, 50, or 100 nM and protein to crRNA ratios of 1:1, 2:1, or 1:2, then monitored at 0, 30, 60, and 90 min. Numbers in each cell indicate mean signal values (n = 3). (G) Fold amplification kinetics at 50 nM Cas13d for each protein to crRNA ratio, calculated as S/B = F/F0, where F and F0 denote fluorescence signals at each time point and at the initial measurement, respectively. The arrow marks the recommended operating condition (50 nM, 1:1) used throughout the study. (H) Fold amplification kinetics at 100 nM Cas13d, plotted as in panel G. (I) Callability expressed as time to result, defined as the earliest time point at which S/B reaches 10 for each condition. NR indicates conditions that did not reach the S/B ≥ 10 threshold within 120 min.

To establish a framework for allele‐specific detection of SNVs, we leveraged the Cas13design platform (http://cas13design.nygenome.org) [37, 38] to generate candidate crRNAs targeting three oncogenic hotspots: *KRAS* G12D, *IDH1* R132C, and *BRAF* V600E (Figure 1A). For each target, an 80‐nt region centered on the variant site served as input. The platform ranked guides by predicted activity on the SNV sequence (Score_SNV), yielding the top ten candidates per target (Figure S2A). To establish a specificity metric, we calculated predicted activities against corresponding wild type alleles (Score_WT) and derived a differential score (ΔScore = Score_SNV − Score_WT). Prioritizing crRNAs with a ΔScore ≥ 0.2 (Figure 1A, Figure S2A) yielded four candidates for *KRAS* G12D, and two each for *IDH1* R132C and *BRAF* V600E (Figure 1B, Figure S2A).

We next evaluated the collateral cleavage activity of these candidates using a fluorescence reporter assay. Among *KRAS* G12D guides, crRNA4 exhibited the largest signal separation between mutant and wild type transcripts, consistent with its high predicted ΔScore of 0.93. In contrast, crRNA1 showed little allelic discrimination despite robust activity, reflecting a lower ΔScore of 0.24 (Figure 1C). For *IDH1* R132C, crRNA1 effectively distinguished mutant from wild type RNA, whereas crRNA2 showed reduced selectivity, in line with their predicted ΔScores of 0.86 and 0.45, respectively (Figure 1D). For *BRAF* V600E, both crRNA1 and crRNA2 exhibited detectable allelic preference, with crRNA2 producing the strongest separation, again matching its higher ΔScore of 0.58 (Figure 1E). Across loci, however, even the best‐ranked guides retained measurable activity on wild type templates, resulting in partial separation between mutant and wild type signals. Consistent with these endpoint measurements, full time‐course trajectories showed discriminatory crRNAs generated steeper fluorescence increases for mutant RNAs, whereas non‐discriminatory guides produced overlapping curves (Figure S2B–D). These results indicated that rational in silico design can generate crRNAs with varying degrees of allelic specificity. Nevertheless, residual wild type activity and limited signal separation observed here are insufficient for precise discrimination between SNV and wild type transcripts, underscoring the need for subsequent engineering to improve selectivity.

To establish a standardized operating point for downstream engineering, we titrated RNP abundance and protein:crRNA stoichiometry (Figure 1F–I). Increasing miniCas13d from 10 to 50 nM accelerated signal accumulation and improved signal‐to‐background (S/B) performance, with a 1:1 protein:crRNA ratio giving the strongest amplification, reaching ∼60‐fold by 90 min and achieving an S/B threshold of 10 within 30 min. At 100 nM Cas13d, fold amplification decreased and non‐equimolar ratios failed to reach the callability threshold within 120 min, consistent with elevated background. We therefore used 50 nM Cas13d assembled at a 1:1 protein:crRNA ratio in subsequent assays.

### 2.2 Rational Engineering of Mismatched crRNAs Enhances Allelic Discrimination

**FIGURE 2:**
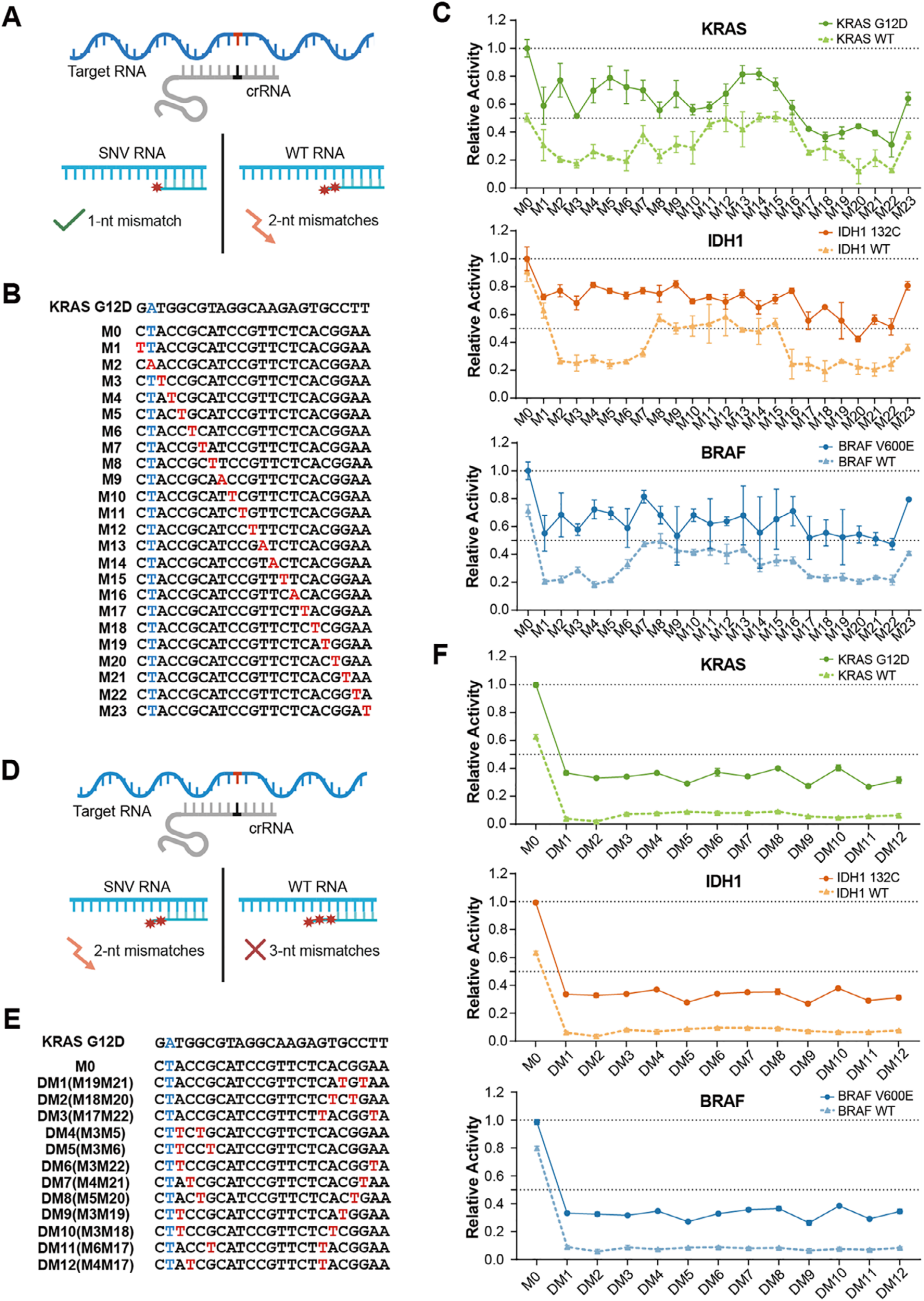
SNV discrimination profiling using engineered mismatch crRNAs. (A) Schematic illustrating the single nucleotide mismatch strategy. Each crRNA was engineered to introduce a single nucleotide mismatch with the SNV target (left), and a double mismatch with the corresponding WT sequence (right). (B) Mismatch variants (M1‐M23) were generated based on *KRAS* G12D crRNA4 by introducing single nucleotide substitutions at each position of the crRNA spacer. Additional engineered mismatches are shown in red, and the nucleotide creating the intrinsic mismatch with the WT sequence at the SNV site is shown in blue. crRNA spacer sequences are shown in the 3′ to 5′ orientation. (C) Cleavage activity of each mismatch variant was assessed against SNV (solid lines) and WT (dashed lines) RNA targets for *KRAS* (green), *IDH1* (orange), and *BRAF* (blue). Fluorescence signals were normalized to the activity of the reference crRNA (M0). Data are presented as mean ± SD (n = 3). (D) Schematic illustrating dual nucleotide mismatch strategy. Each crRNA was engineered to introduce two mismatches with SNV target (left), and three mismatches with corresponding WT sequence (right). (E) Dual mismatch crRNA variants (DM1‐DM12) were generated based on *KRAS* G12D crRNA4 by combining selected single mismatch positions from M1‐M23 set. Additional engineered mismatches are shown in red, and the nucleotide creating the intrinsic mismatch with the WT sequence at the SNV site is shown in blue. crRNA spacer sequences are shown in the 3′ to 5′ orientation. (F) Cleavage activity of dual mismatch crRNAs against SNV (solid lines) and WT (dashed lines) RNA targets for *KRAS* (green), *IDH1* (orange), and *BRAF* (blue). Fluorescence signals were normalized to the activity of the reference crRNA (M0). Data are presented as mean ± SD (n = 3).

To enhance allelic specificity, we systematically engineered crRNAs by introducing defined mismatches across the spacer region (Figure 2A,B). For each SNV locus, the most selective crRNA from the initial screen was selected as the parental M0 reference: *KRAS* G12D crRNA4, *IDH1* R132C crRNA1, and *BRAF* V600E crRNA2. Using each M0 guide as a template, we generated 23 single mismatch variants, designated M1 through M23, by introducing a single nucleotide substitution at each position across the 23‐nt spacer. Each design introduced one mismatch relative to the SNV template and two mismatches relative to its wild type counterpart, with one located at the engineered position and the other at the variant site (Figure 2B; Figure S3A,B).

Across all three loci, miniCas13d showed pronounced position dependent sensitivity to mismatches, consistent with functionally constrained segments within the spacer that disproportionately influence target engagement and nuclease activation. Two spacer windows, M2‐M6 and M17‐M22, consistently produced the greatest allelic separation, with wild type activity strongly suppressed while SNV activity remained partially preserved at approximately 0.4–0.8 relative to the M0 reference (Figure 2C). In contrast, mismatches outside these windows either reduced activity similarly for both templates or had little effect, resulting in minimal discrimination. Across the three loci tested, these data identify M2‐M6 and M17‐M22 as key positions for engineering allelic selectivity and provide practical heuristics for crRNA design.

To place these mismatch positions in a mechanistic context, we mapped them onto an AlphaFold3‐predicted structural model of the miniCas13d‐crRNA‐target RNA ternary complex (Figure S3E). Spacer positions are numbered sequentially from the distal end toward the direct repeat (DR). Under this convention, the M2‐M6 segment lies at the distal spacer end and may serve as an initial target‐binding or nucleation window. This region is thought to contribute to early target capture, making it sensitive to local target accessibility and to a small number of mismatches. Consequently, mismatches introduced within this window would likely impair or markedly slow binding, hindering progression to an activated state. In contrast, M17‐M22 resides in the proximal spacer region closer to the DR and localizes adjacent to the HEPN catalytic core, consistent with an activation gate or nuclease‐switch window. Following initial capture, pairing quality in this segment may influence whether guide‐target base pairing can efficiently propagate along the duplex to trigger HEPN‐coupled conformational rearrangements that promote entry into a high‐activity cleavage state [18, 30, 39]. Accordingly, mismatches in M17‐M22 may permit partial binding but substantially reduce activation and collateral cleavage. This spatial separation suggests that mismatches in the two windows perturb distinct checkpoints of early target engagement and catalytic activation. As a result, when an engineered mismatch is combined with the inherent SNV mismatch, the wild type template is preferentially penalized, thereby sharpening single‐nucleotide discrimination.

We next generated a panel of double mismatch (DM) guides by pairing mismatch positions within and between the two mismatch‐sensitive regions, M2‐M6 and M17‐M22, yielding 12 predefined DM designs (DM1‐DM12) (Figure 2D,E; Figure S3C,D). These designs further improved discrimination but frequently at the expense of catalytic activity (Figure 2F). Specifically, DM10 (M3M18) preserved 45% activity on the mutant target while reducing wild type recognition to <10%. Other variants, including DM5 (M3M6), DM9 (M3M19), and DM11 (M6M17), nearly abolished wild type cleavage but attenuated mutant activity to 20%–40% of baseline. Thus, double mismatches markedly suppress wild type recognition but at the cost of catalytic efficiency, indicating that mismatch engineering improves allelic discrimination yet cannot fully overcome the specificity‐activity trade‐off. This limitation underscores the need for additional strategies to achieve reliable single nucleotide resolution.

The positional sensitivity was consistent across *KRAS* G12D, *IDH1* R132C, and *BRAF* V600E assays. In all three targets, engineered mismatches placed within the M2‐M6 or M17‐M22 windows produced the strongest separation between SNV and wild type reporter signals. This same positional preference held for both single‐mismatch and double‐mismatch guide designs, indicating that these two windows represent spacer segments that are particularly mismatch‐sensitive for the effector. We further tested this positional rule on two additional oncogenic SNVs, *EGFR* L858R and *PIK3CA* H1047R, and again observed optimal discrimination when mismatches were positioned within M2‐M6 or M17‐M22, supporting its applicability across distinct local sequence contexts (Figure S4A,B). Collectively, these results support a generalizable guide‐design strategy for oncogenic SNVs: in the presence of an intrinsic variant mismatch, introducing an additional synthetic mismatch within one of these two windows selectively suppresses the wild type template and sharpens single nucleotide discrimination.

### 2.3 Sensitivity Analysis of miniCas13d for SNV Detection

**FIGURE 3:**
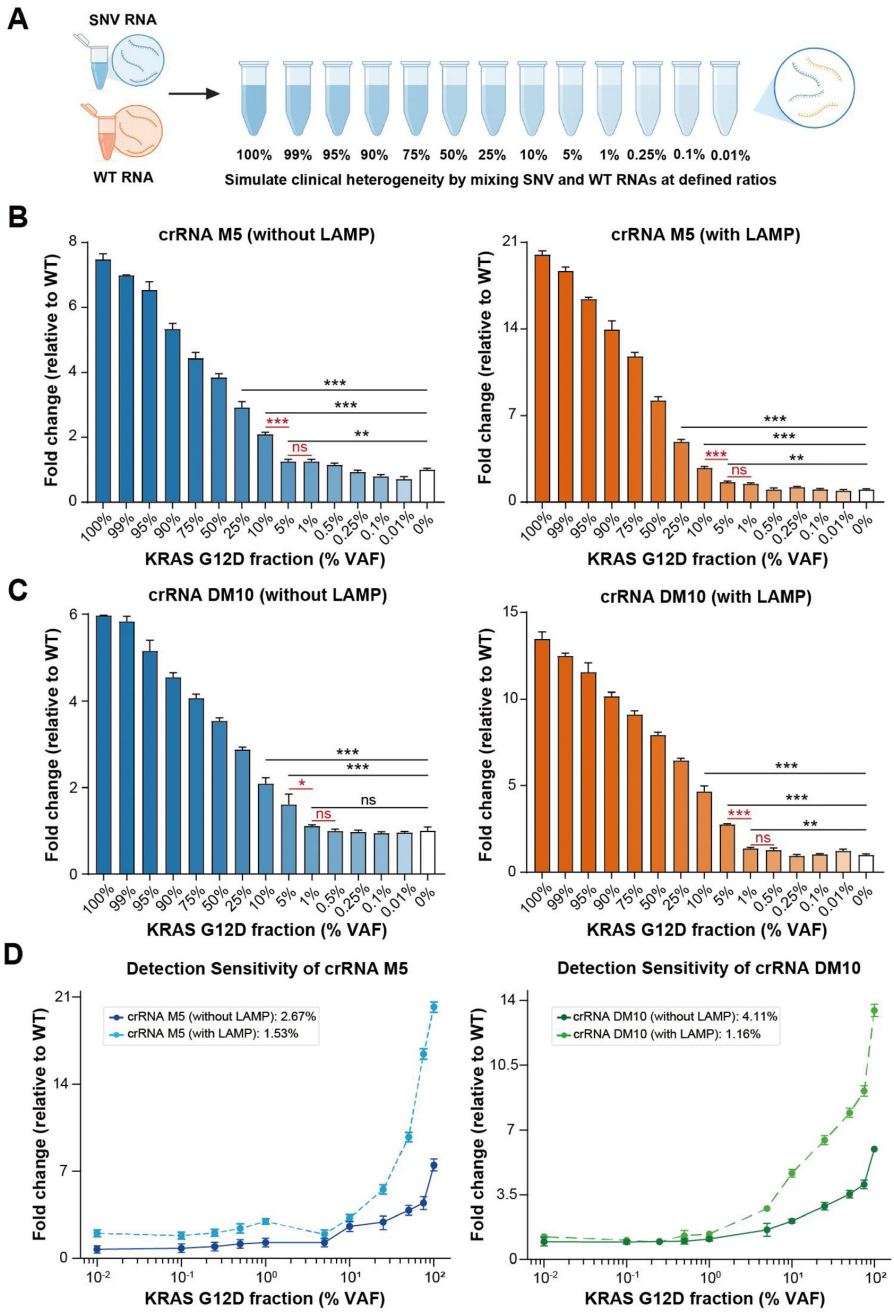
Sensitivity evaluation of SNV detection by miniCas13d across a gradient of mutant allele fractions. (A) Schematic illustrating the experimental design for SNV detection sensitivity. Synthetic *KRAS* G12D (SNV) and wild type (WT) RNAs were mixed at defined ratios to generate samples with mutant allele fractions ranging from 100% to 0.01%, followed by Cas13d based detection. (B,C) Bar plots showing fluorescence fold change relative to WT control (0% G12D) across dilution series for crRNA M5 (B) and DM10 (C), measured without (left) and with (right) LAMP amplification. Data are presented as mean ± SD (n = 3). Statistical significance was assessed by independent samples *t*‐tests comparing each SNV dilution to WT control. * *p* < 0.05; ** *p* < 0.01; *** *p* < 0.001; ns, not significant. (D) Line plots showing fluorescence fold change relative to WT across *KRAS* G12D RNA dilutions for crRNA M5 (left) and DM10 (right), without (solid lines) and with (dashed lines) LAMP. Estimated fractional limits of detection (fLoD, %) based on EP17‐A2 analysis are indicated. Data are shown as mean ± SD (n = 3).

To evaluate the sensitivity of miniCas13d for SNV detection, we employed *KRAS* G12D as a representative mutation and prepared RNA mixtures with defined variant allele frequencies (VAFs) ranging from 100% to 0.01% (Figure 3A). Two crRNA guides were tested: M5, containing a single mismatch at position 5, and DM10, featuring dual mismatches at positions 3 and 18. Each guide was assessed under conditions with or without pre‐amplification using loop‐mediated isothermal amplification (LAMP). Without amplification, the M5‐guided miniCas13d system exhibited a robust response at high mutant fractions, with fluorescence reaching approximately eightfold above WT at 100% VAF (Figure 3B). Signal intensity declined progressively with decreasing mutant content. Samples containing 5% mutant RNA showed a statistically significant elevation over WT, but the signal plateaued thereafter, with no significant difference detected between 5% and 1% variants, reflecting limited resolution at low allele frequencies. LAMP pre‐amplification substantially increased fluorescence across all VAFs, yielding up to 20‐fold signal at 100% VAF, yet resolution between 5% and 1% remained limited, indicating marginal sensitivity gain at low frequency detection. The DM10 guide displayed a similar trend. Under direct detection, it generated strong signals for high VAFs and maintained clear discrimination from WT for VAFs ranging from 95% to 5% (Figure 3C; Figure S5A,B). However, the ability to differentiate became progressively weaker as the mutant content dropped to 1% or below. Incorporating LAMP further increased fluorescence output, reaching up to 14‐fold over WT baseline at 100% VAF. The amplified reactions exhibited a smooth and nearly monotonic decline in fold change from 95% down to 5% VAF, with signals across this range remaining well resolved. However, at VAFs of 1% and lower, signal separation diminished markedly, and no statistically significant difference was observed between 1% and 0.5% VAF groups.

To quantitatively characterize detection sensitivity across mutant allele frequencies, we plotted fluorescence fold change relative to WT as a function of *KRAS* G12D VAF (Figure 3D) and determined fractional limit of detection (fLoD) using CLSI EP17‐A2 guidelines (Table 2) [40]. Without pre‐amplification, the miniCas13d system showed limited responsiveness at low mutant abundance, with both M5‐ and DM10‐guided detection producing minimal signal elevation below 5% VAF and appreciable fold change only above this threshold. Isothermal amplification markedly boosted overall signal intensity and broadened the dynamic range, especially at mutant fractions ≥10%, but the distinction between WT and 1% mutant RNA remained modest (Figure 3D). Consistent with these observations, fLoD values confirmed that LAMP pre‐amplification consistently lowered detection thresholds for both guide designs, although most of the visual signal separation remained concentrated at higher VAFs. For M5 crRNA, fLoD decreased from 2.67% to 1.53% with LAMP. Similarly, DM10 crRNA fLoD dropped from 4.11% to 1.16%. Despite these improvements, reliably differentiating ultra‐low abundance mutations from WT background remained challenging, highlighting a practical sensitivity limitation under the current assay conditions.

**TABLE 2:** CLSI EP17‐A2 analytical sensitivity metrics for LwaCas13a and engineered RfxCas13d protein systems.

miniCas13d	Blank_ mean_slope	Blank_ SD_slope	LoB_slope	SD_low_slope	LoD_slope	b̂	fLoD (%)
**M5**	10.211	0.533	11.088	0.825	12.445	0.838	2.667
**M5+LAMP**	20.415	1.071	22.176	1.705	24.981	2.985	1.529
**DM10**	9.013	0.676	10.126	0.275	10.577	0.381	4.108
**DM10+LAMP**	16.154	0.727	17.349	0.818	18.695	2.193	1.159
**LwaCas13a**	**Blank_** **mean_slope**	**Blank_** **SD_slope**	**LoB_slope**	**SD_low_slope**	**LoD_slope**	**b̂**	**fLoD (%)**
**M5**	61.915	0.547	62.815	1.645	65.650	1.514	2.723
**M5+LAMP**	50.415	2.276	54.159	3.568	60.564	4.114	2.671
**Y119‐RBD7**	**Blank_** **mean_slope**	**Blank_** **SD_slope**	**LoB_slope**	**SD_low_slope**	**LoD_slope**	**b̂**	**fLoD (%)**
**M5**	103.892	1.864	106.958	14.653	131.061	17.838	1.573
**M5+LAMP**	322.365	9.305	337.672	27.245	382.490	36.321	1.555
**DM10**	55.688	1.455	58.081	0.958	59.658	6.512	0.610
**DM10+LAMP**	211.356	5.747	220.809	25.998	263.577	31.491	1.658
**N47‐RBD7** **+Y119‐RBD6**	**Blank_** **mean_slope**	**Blank_** **SD_slope**	**LoB_slope**	**SD_low_slope**	**LoD_slope**	**b̂**	**fLoD (%)**
**M5**	292.095	5.976	301.926	27.132	346.558	21.322	2.554
**M5+LAMP**	524.627	9.958	541.008	25.645	583.194	39.363	1.488
**DM10**	132.283	0.475	133.064	2.124	136.558	7.276	0.588
**DM10+LAMP**	445.356	15.543	470.924	29.849	520.026	32.451	2.301

**Table footnote. Slope**: the initial reaction rate (ΔRFU·min^−1^) measured in the early linear regime. **Blank_mean_slope** and **Blank_SD_slope** are the mean and SD of slope values from 0% VAF replicates. **LoB_slope (limit of blank)** = Blank_mean_slope + 1.645×Blank_SD_slope (95th percentile of blank). **SD_low_slope** is the SD of slope values from the low‐level sample used for LoD estimation. **LoD_slope (limit of detection)** = LoB_slope + 1.645×SD_low_slope (95% detection probability assumption). **b̂** is the estimated low‐range sensitivity coefficient (slope of the regression of Δslope = slope − Blank_mean_slope versus VAF(%), fitted over the specified low‐VAF window). **fLoD (%)**: the fractional LoD expressed as VAF, computed as fLoD = (LoD_slope − Blank_mean_slope)/b̂.

### 2.4 Structure‐Guided Engineering of miniCas13d With Rationally Positioned RNA Binding Domains

**FIGURE 4:**
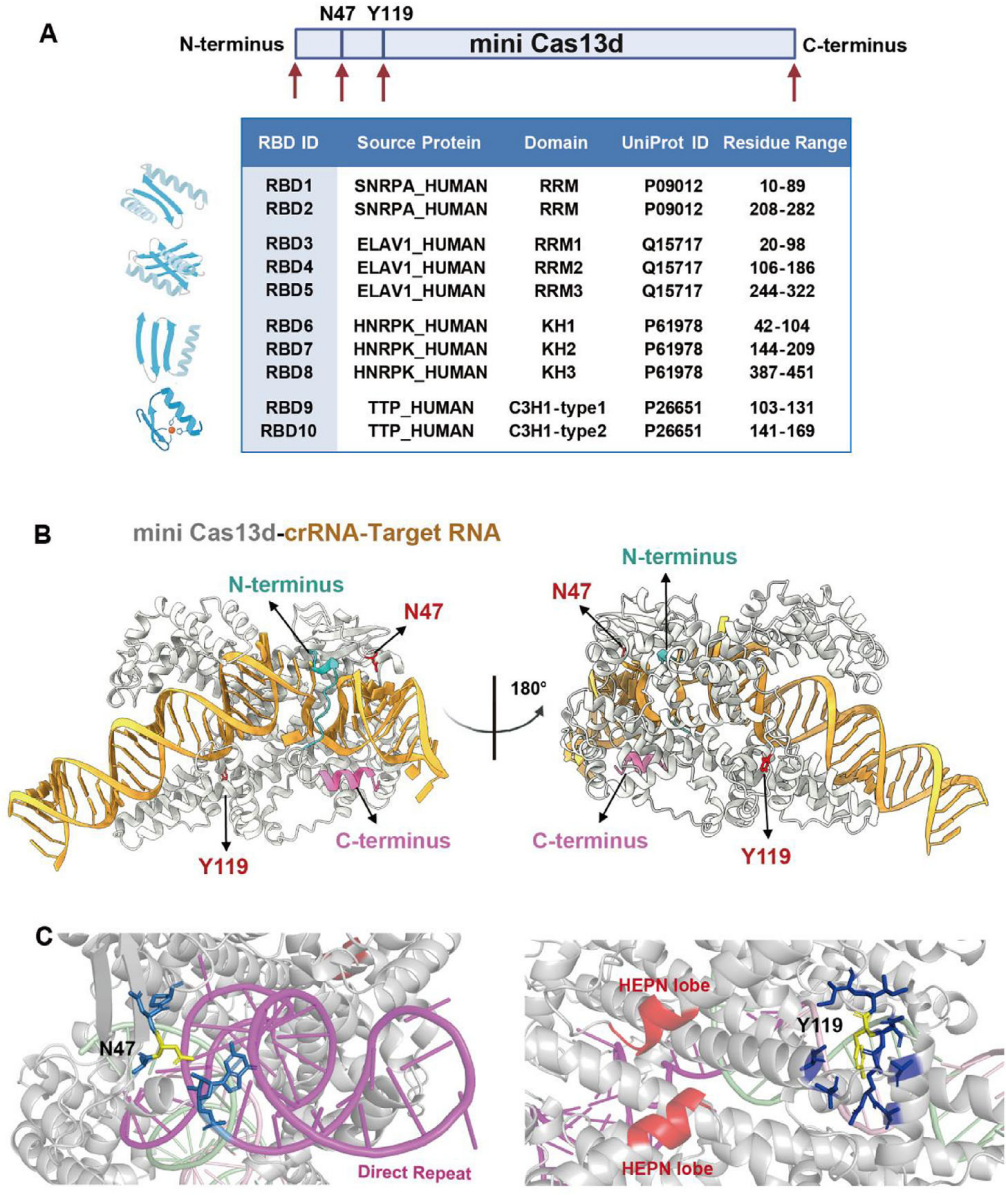
Rational design and structural positioning of RNA binding domains in engineered miniCas13d. (A) Schematic of miniCas13d highlighting four engineered insertion sites (N‐terminus, C‐terminus, N47, and Y119) indicated by red arrows. Ten candidate RNA binding domains (RBD1‐RBD10) were selected from human RNA binding proteins. The domain type, UniProt ID, and residue range are listed. (B) Structural model of the miniCas13d‐crRNA‐target RNA ternary complex, predicted by AlphaFold3. The engineered insertion sites (N47 and Y119) are highlighted in red, while N‐terminus and C‐terminus are shown in cyan and pink, respectively. (C) Local structural context of two internal fusion sites. N47 resides in the N terminal region of miniCas13d, immediately adjacent to the crRNA direct repeat (DR) scaffold and the DR proximal interaction surface. Y119 is located in a solvent exposed region near the RNA binding cleft and adjacent to the catalytic HEPN lobe, in proximity to the HEPN core motifs (R138‐H143 and R610‐H615).

Mismatch‐engineered crRNAs improved allele discrimination but often reduced overall signal, most noticeably when target input was low. We therefore asked whether signal output could be increased without relaxing the mismatch‐driven specificity by engineering the miniCas13d scaffold to better engage RNA substrates. To do this, we fused compact heterologous RNA binding domains (RBDs) to miniCas13d at surface‐accessible positions that were unlikely to disrupt the core fold or obstruct the RNA binding cleft and HEPN nuclease lobe (Figure 4A) [29].

Fusion‐site selection was guided by analysis of the AlphaFold3‐predicted miniCas13d‐crRNA‐target RNA ternary model in the context of available EsCas13d cryo‐EM structures [18, 30, 41]. We selected fusion sites by balancing structural permissiveness with functional proximity. Specifically, we prioritized solvent‐exposed termini or surface segments predicted to tolerate insertion without perturbing the overall architecture. Additionally, we targeted locations near the crRNA handle (DR) interface, the guide‐target duplex path, or the HEPN nuclease lobe, where added RNA contact could plausibly influence target docking or activation. This framework identified four sites: the N‐ and C‐ termini, an internal site (N47) within a surface‐exposed segment adjacent to the DR‐proximal region in our model, and a second internal site (Y119) positioned near the RNA binding cleft and proximal to the catalytic HEPN lobe (HEPN motifs R138‐H143 and R610‐H615) (Figure 4B,C).

We then assembled a modular panel of ten human RBDs comprising five RRMs, three KH fragments, and two zinc‐finger modules, chosen for compact size and their ability to bind single‐stranded RNA with modest sequence preferences. We fused each domain at each of the four sites to generate 40 miniCas13d‐RBD variants. To triage designs for gross incompatibilities, we used AlphaFold3 predictions to assess model plausibility and the confidence of the RNA‐bound architecture. pTM and ipTM were used as confidence metrics for global topology and interface placement. Across the library, ternary‐complex predictions yielded pTM scores of 0.65–0.76 and ipTM scores of 0.50–0.71, consistent with most fusions being compatible with the miniCas13d scaffold, while a subset showed reduced interface confidence suggestive of localized changes at the RNA‐protein interface introduced by the insertion.

By integrating AlphaFold3‐based structural modeling, RNA proximity analysis, and ipTM‐guided interface evaluation, we shortlisted eight miniCas13d‐RBD variants for experimental validation: N‐RBD6, C‐RBD2, C‐RBD4, Y119‐RBD3, Y119‐RBD6, Y119‐RBD7, N47‐RBD3, and N47‐RBD7 (Figure S6A–H). These variants showed favorable global model confidence (pTM: 0.66–0.76) and moderate‐to‐high interfacial confidence (ipTM: 0.56–0.71). To further combine functionally advantageous modules and evaluate potential synergistic effects, we additionally engineered a dual‐RBD Cas13d variant by fusing RBD7 at N47 and RBD6 at Y119 (N47‐RBD7+Y119‐RBD6). All eight single‐RBD variants were successfully expressed and purified in *Escherichia coli*, as confirmed by SDS‐PAGE analysis (Figure S6I). The purified proteins exhibited consistent molecular weights in expected range (∼75 kDa), indicating proper folding and construct integrity. The dual‐RBD Cas13d variant was similarly expressed and purified, with comparable yield and purity.

### 2.5 Engineered miniCas13d‐RBD Variants Enhance SNV Recognition Activity and Specificity

**FIGURE 5:**
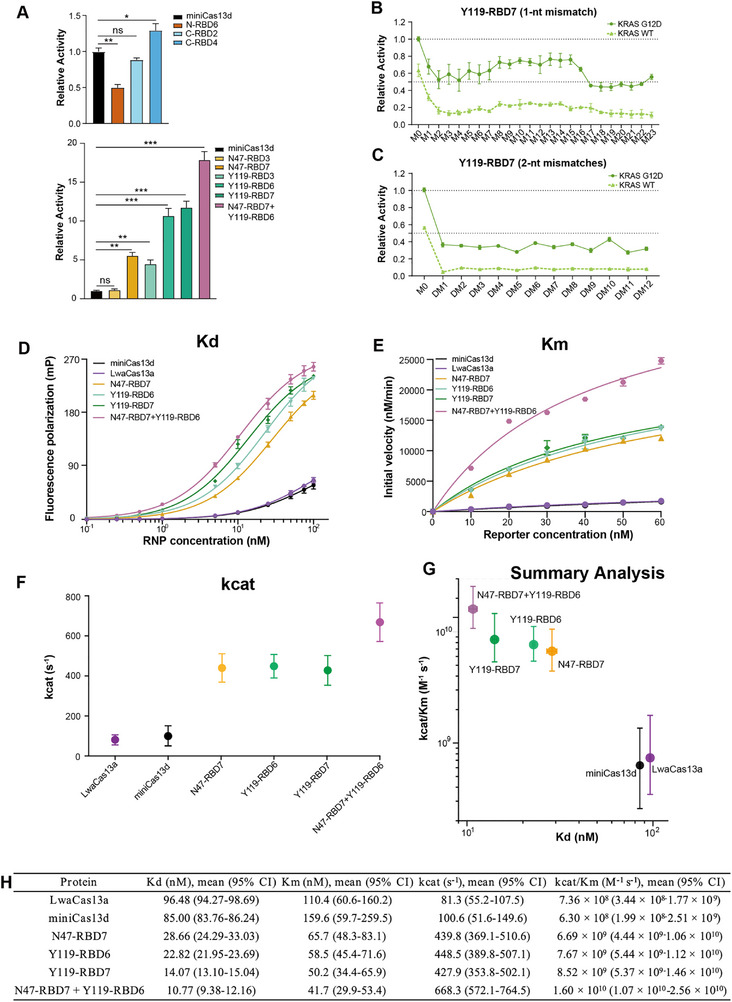
Evaluation of engineered miniCas13d variants for SNV detection. (A) Cleavage activity of miniCas13d variants with RBDs inserted at terminal positions in the top panel and at internal positions in the bottom panel. Fluorescence was measured at 90 min and normalized to miniCas13d. Black represents miniCas13d; orange and blue indicate N‐ and C‐terminal fusions, respectively; yellow and green indicate insertions at N47 and Y119 sites; pink represents dual‐RBD variant N47‐RBD7+Y119‐RBD6. Data are presented as mean ± SD (n = 3). Statistical significance was assessed using independent samples *t*‐tests. * *p* < 0.05; ** *p* < 0.01; *** *p* < 0.001; ns, not significant. (B,C) Mismatch discrimination profiling of Y119‐RBD7 using crRNAs containing either single nucleotide mismatches (B, M1‐M23) or dual mismatches (C, DM1‐DM12). Relative cleavage activity against *KRAS* G12D (solid lines) and WT RNA (dashed lines) was quantified by fluorescence and normalized to the reference crRNA M0. Data are presented as mean ± SD (n = 3). (D) Fluorescence polarization binding curves used to derive *K*_d_ for miniCas13d, LwaCas13a, and RBD‐fusion Cas13d variants. Fluorescence polarization was measured at equilibrium across a titration series of cognate Cas13‐crRNA RNPs and fitted to a single site binding model. Data points are shown with 95% confidence intervals (n = 3). (E) Reporter saturation analysis used to determine *K*_m_ and *V*_max_. Initial velocity was plotted as a function of reporter concentration and fitted to the Michaelis‐Menten equation for each variant. Data points are shown with 95% confidence intervals (n = 3). (F) Apparent turnover numbers (*k*_cat_) calculated from *V*_max_ normalized by enzyme concentration under the matched steady‐state conditions in panel E. Values are shown with 95% confidence intervals. (G) Integrated summary plotting catalytic efficiency (*k*_cat_/*K*_m_) versus *K*_d_ for miniCas13d, LwaCas13a, and RBD‐fusion Cas13d variants. (H) Tabulated thermodynamic and kinetic parameters for each variant, shown as mean with 95% confidence intervals.

To evaluate the functional impact of RBD insertion, we quantified the cleavage activity of miniCas13d‐RBD variants using fluorescence assays guided by the reference guide M0 targeting *KRAS* G12D. Among terminal insertions, N‐RBD6 exhibited only about half of the parental miniCas13d activity, whereas miniCas13d C‐RBD2 and C‐RBD4 retained or slightly enhanced activity levels (Figure 5A, upper panel; Figure S7A). Structural modeling with AlphaFold3 revealed that both termini of miniCas13d are located near the 3′ end of the crRNA, a region stabilized by extensive native protein‐RNA contacts, likely limiting the functional gain achievable through terminal RBD fusion. In contrast, insertion of RBDs at internal sites led to substantial improvements in target cleavage. Y119‐RBD7 emerged as the top‐performing single‐RBD variant, with cleavage activity increased by 12.6‐fold compared to parental miniCas13d. Y119‐RBD6, N47‐RBD7, and Y119‐RBD3 also demonstrated strong enhancements, with activity elevated by 4.5‐ to 9.5‐fold, while N47‐RBD3 had moderate effects. The dual‐RBD variant N47‐RBD7+Y119‐RBD6 produced the strongest enhancement, achieving a 17.4‐fold increase in cleavage activity, and showed the highest catalytic output among tested variants (Figure 5A, lower panel; Figure S7A). These data indicate that combining RBDs can substantially boost apparent cleavage activity.

We then profiled Y119‐RBD7 using a comprehensive crRNA mismatch library targeting *KRAS* G12D, *IDH1* R132C, and *BRAF* V600E to evaluate whether RBD fusion alters sequence specificity. The library included single nucleotide mismatches spanning all spacer positions (M1‐M23) and selected double mismatch combinations (DM1‐DM12) (Figure 5B,C; Figure S7B,C). Despite marked enhancement in overall activity, Y119‐RBD7 maintained a mismatch sensitivity pattern that closely resembled that of parental miniCas13d, with positional effects largely preserved. Consistent with parental miniCas13d, Y119‐RBD7 exhibited a clear gradient of mismatch tolerance. Two regions, M2‐M6 and M17‐M22, produced the strongest separation between mutant and wild type signals: wild type cleavage was strongly reduced, while mutant activity was only partially reduced, typically retained at 50%–70% of matched reference. Positions flanking these regions incurred intermediate penalties. By contrast, mismatches within central segment (M8‐M16) were broadly tolerated by both mutant and wild type targets. Although mutant activity remained high, elevated wild type signal limited discriminatory power at these positions (Figure 5B). Double mismatch profiling of Y119‐RBD7 further confirmed this positional dependence. Guides incorporating two mismatches within the separation bands M2‐M6 and M17‐M22 yielded the greatest specificity, with mutant cleavage remaining detectable and wild type activity reduced to background levels (Figure 5C). For instance, DM10 (M3M18) retained approximately 47% of matched activity on the mutant target with negligible wild type cleavage. This positional rule extended to both *IDH1* R132C and *BRAF* V600E, indicating broad applicability across SNV contexts (Figure S7B,C). These findings show that Y119‐RBD7 preserves positional selectivity architecture of parental miniCas13d while operating at a significantly elevated activity baseline.

For deeper mechanistic insight, we investigated whether internal RBD insertions elevate signal output by enhancing target engagement or accelerating apparent collateral turnover. We decoupled these effects by quantifying equilibrium activator binding with a nuclease‐resistant, FAM‐labeled 2′‐O‐methyl probe, and assessing catalytic capacity by fitting initial‐rate measurements to a Michaelis‐Menten kinetics. For the single‐RBD variants, insertions resulted in both improved binding affinity and enhanced apparent collateral catalysis (Figure 5D–H). Compared to the parental miniCas13d, the *K*_d_ improved from 85 nM to 14–29 nM, the *K*_m_ declined from approximately 160 nM to 50–66 nM, and the *k*_cat_ increased from about 101 to 428–449 s^−1^. These shifts produced a 10‐ to 14‐fold increase in catalytic efficiency relative to miniCas13d. Although our structural models predicted that the N47 and Y119 sites might independently govern distinct steps, the biochemical data indicate that insertions at either site improve both binding affinity and catalytic turnover. This suggests that stabilizing either the crRNA handle or the catalytic cleft may favor a more productive, activated conformation of the Cas13d ternary complex. The dual‐RBD variant further amplified these trends. It improved the *K*_d_ to 11 nM, lowered the *K*_m_ to 42 nM, and increased the *k*_cat_ to 668 s^−1^. These cooperative shifts culminated in a 25‐fold enhancement in catalytic efficiency (Figure 5D–H). The combined binding and kinetic data demonstrate that RBD fusions concurrently improve target engagement and collateral turnover within the reporter concentration range tested.

**FIGURE 6:**
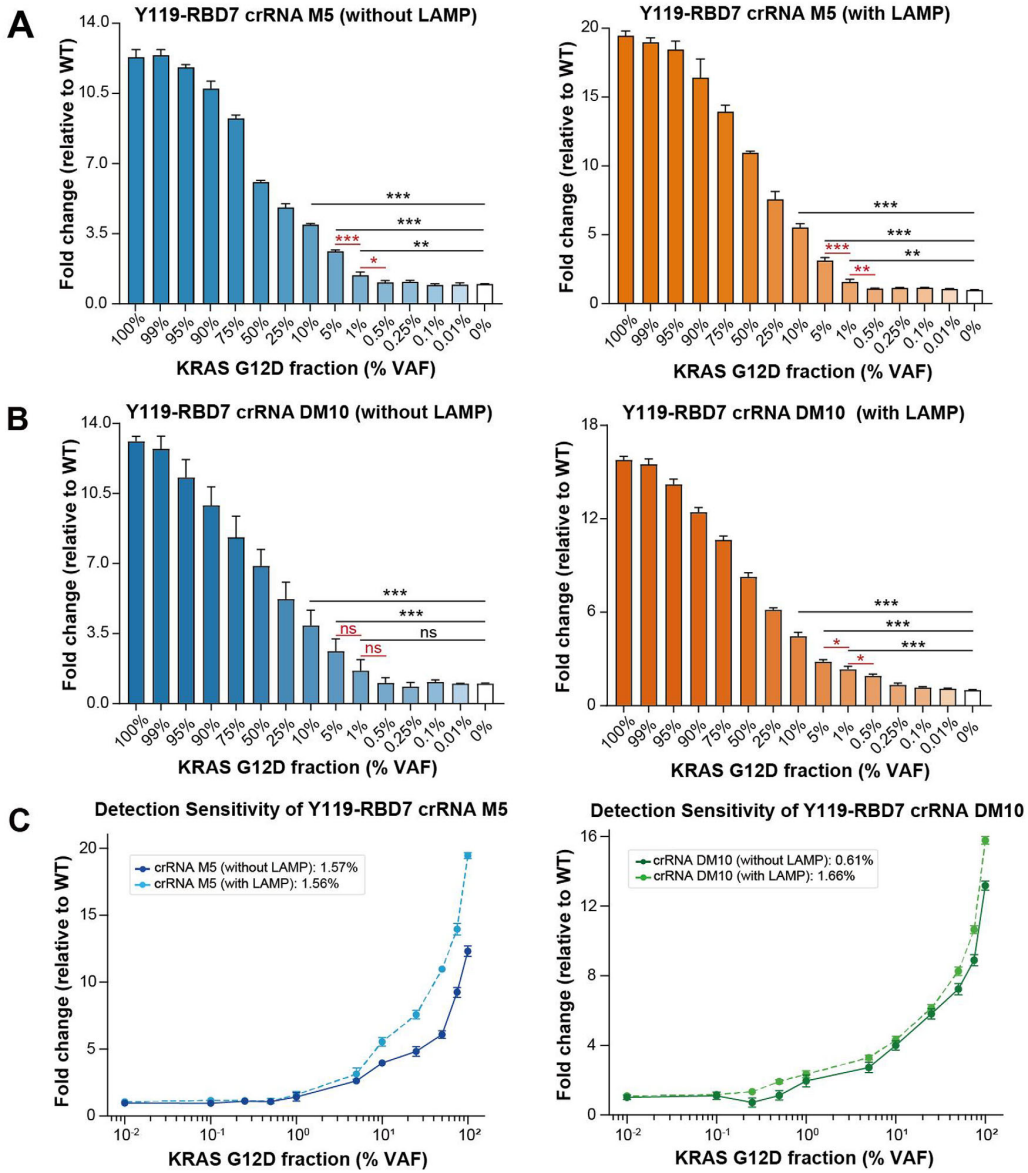
Performance of Y119‐RBD7 for RNA SNV detection. (A,B) Detection sensitivity of Y119‐RBD7 variant using engineered mismatch crRNAs M5 (A) and DM10 (B) across a *KRAS* G12D dilution series. Fluorescence signals were measured without LAMP (left) and with LAMP (right) and expressed as fold change relative to WT control (0% G12D). Data are presented as mean ± SD (n = 3). Statistical significance was assessed using independent samples *t*‐tests. * *p* < 0.05; ** *p* < 0.01; *** *p* < 0.001; ns, not significant. (C) Line plots showing fold changes in fluorescence relative to WT across *KRAS* G12D RNA dilutions for crRNA M5 (left) and DM10 (right), without LAMP (solid lines) and with (dashed lines) LAMP. Estimated fractional limits of detection (fLoD, %) derived from EP17‐A2 analysis are indicated. Data are presented as mean ± SD (n = 3).

We next evaluated diagnostic performance by measuring analytical sensitivity across a 0%–100% *KRAS* G12D mutant‐fraction series. First, we benchmarked three high‐performing single‐RBD variants (Y119‐RBD7, N47‐RBD7, and Y119‐RBD6) using the mismatch‐optimized guides M5 and DM10. Under amplification‐free conditions, these single variants achieved fLoDs ranging from 1.57%–2.04% for M5, and 0.61%–0.90% for DM10 (Figure 6A–C and Figures S8–S10). Time‐course kinetics confirmed that the addition of LAMP broadened the dynamic range but did not uniformly improve fractional sensitivity. For instance, LAMP shifted the fLoD range to 1.56%–2.03% for M5, and worsened it to 1.66%–2.41% for DM10 (Figure 6C; Figure S8–S10). We then asked whether combining two distinct insertion sites could yield additive diagnostic gains (Figures S8 and S9). Evaluating the dual‐RBD variant (N47‐RBD7+Y119‐RBD6) established that combinatorial insertion benefits are conditional rather than universal (Figure S9A–C). Consistent with its superior catalytic efficiency, the dual‐RBD variant outperformed all single‐RBD variants under the most stringent, amplification‐free workflow with the DM10 guide, pushing the fLoD down to 0.59%. However, it did not universally dominate across all formats, yielding fLoDs of 2.55% for M5 without amplification and 2.30% for DM10 with LAMP (Figure S9A–C).

Given the conditional benefits of target pre‐amplification, we investigated how LAMP alters blank background behavior and EP17‐A2 decision thresholds in the low‐VAF regime [42]. Notably, the impact of LAMP depended on mismatch number. For the single‐mismatch M5 guide, LAMP improved fLoD in specific variant contexts. Conversely, for the double‐mismatch DM10 guide, LAMP did not improve sensitivity under our conditions. Instead, it systematically worsened the fLoD across all variants. This is consistent with amplification increasing both target‐derived signal and residual background that remains detectable with this guide (Figure S11A–D). Within the EP17‐A2 workflow, we quantified this effect using the initial fluorescence slope as the analytical signal, computed LoB and LoD_slope from WT‐only replicates, and converted LoD_slope to fractional LoD using the slope‐mutant‐fraction calibration (b̂) from the dilution series. For DM10, LAMP increased the blank slope and LoD_slope thresholds across all engineered Cas13d variants. For instance, with the Y119‐RBD7 variant, the blank slope surged from 55.7 to 211.4 ΔRFU min^−1^, driving the LoD_slope from 59.7 to 263.6 ΔRFU min^−1^ (Table 2). Similar severe background inflation was observed for N47‐RBD7, Y119‐RBD6, and the dual‐RBD variant.

We further contextualized our platform's diagnostic potential by conducting a head‐to‐head comparison against the established LwaCas13a effector at the *KRAS* G12D locus. Using two crRNAs that satisfy the LwaCas13a protospacer flanking site requirement, one designed via SHERLOCK rules and another sequence‐matched to our Cas13d guide, we observed modest mutant‐to‐WT signal separations of 1.71‐fold and 1.34‐fold at the 90 min endpoint, respectively (Figure S10E,F). Subsequent low‐VAF quantification using a mismatch‐optimized LwaCas13a guide under the standardized EP17‐A2 workflow established baseline fLoDs of 2.723% without amplification and 2.671% with LAMP (Figure S10G, H; Table 2). Under the same amplification‐free conditions, engineered Cas13d variants paired with double‐mismatch guide DM10 exhibited better sensitivity than LwaCas13a, with fLoDs ranging from 0.59%–0.90% for dual‐RBD variant and its parent single variants. These head‐to‐head results show that engineered Cas13d outperformed LwaCas13a in an amplification‐free workflow, whereas the effect of LAMP differs with the number of introduced mismatches.

### 2.6 Performance Evaluation of Modular miniCas13d‐RBD Platforms Across Amplification Conditions

**FIGURE 7:**
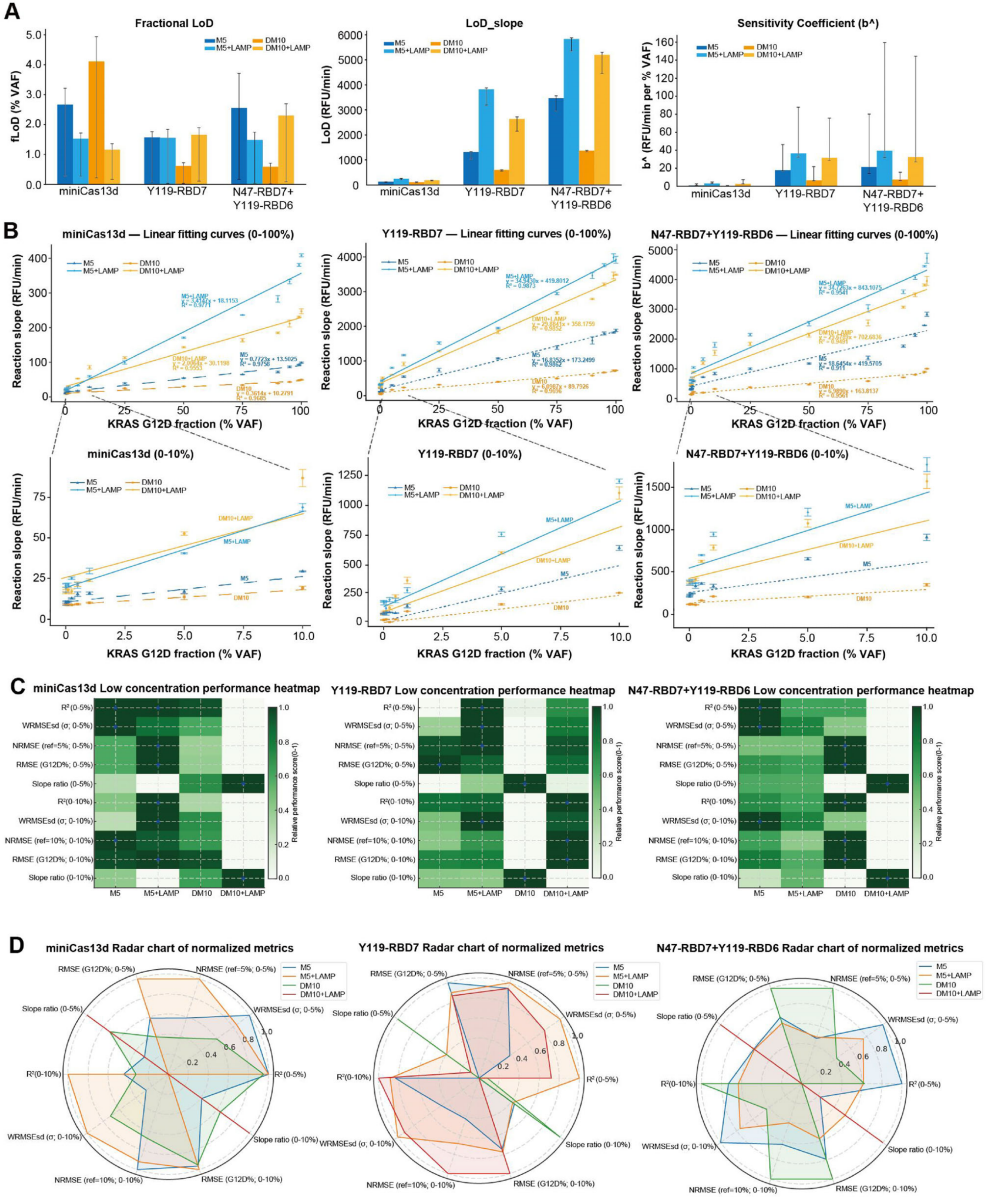
Analytical characterization and comparative performance of engineered miniCas13d platforms. (A) Summary of performance metrics across miniCas13d backbones and assay modes. Bars report fLoD, LoD_slope, and sensitivity coefficient b̂ for miniCas13d, Y119‐RBD7, and N47‐RBD7+Y119‐RBD6 under M5, M5+LAMP, DM10, and DM10+LAMP. All metrics were computed following CLSI EP17‐A2. Error bars denote 95% confidence intervals. (B) Linear standard curves fitted from mean slope values across 0%–100% SNV frequencies for each Cas13d variant under four assay modes. Lower panels show magnified views of 0%–10% low frequency range. (C) Low fraction performance heatmaps comparing assay modes within each backbone using R^2^, WRMSEsd, NRMSE, RMSE%, and slope ratio for the 0%–5% and 0%–10% ranges. Darker green indicates better performance. The best performing assay mode in each row is marked with a blue asterisk. (D) Radar plots summarizing the same ten metrics. Each axis is normalized from 0 to 1, with 1 indicating the best performance. Colored lines indicate assay modes: blue, M5; orange, M5+LAMP; green, DM10; red, DM10+LAMP. Larger areas reflect more balanced overall performance.

We quantified platform performance within a unified analysis framework to characterize how crRNA architecture and isothermal pre‐amplification jointly shape single nucleotide detection performance. We evaluated three Cas13d configurations: the unmodified miniCas13d, the single‐RBD variant Y119‐RBD7, and the dual‐RBD Cas13d variant N47‐RBD7+Y119‐RBD6. Each was paired with either a single mismatch guide M5 or a double mismatch guide DM10 targeting *KRAS* G12D, and tested under direct detection and LAMP coupled workflows. Analytical sensitivity was assessed according to CLSI EP17‐A2, reporting fLoD, LoD_slope and sensitivity coefficient b̂ (Figure 7A). Quantitative behavior in low VAF regime (0%–5%, 0%–10%) was evaluated primarily by deviation from global 0%–100% calibration, expressed as normalized root mean square error (NRMSE), and by local‐to‐global slope ratio, which reflects the relative low‐range response compared with the full‐range calibration (Figure 7B–D). Precision and dispersion, along with absolute percent error, were further summarized by weighted root mean square error standard deviation (WRMSEsd) and root mean square error percentage (RMSE%) (Table 3).

**TABLE 3:** Low frequency quantitative performance of miniCas13d and engineered variants across 0%–5% and 0%–10% VAF ranges.

miniCas13d	R2 (0%–5%)	WRMSEsd (σ; 0%–5%)	NRMSE (%) (ref = 5%; 0%–5%)	RMSE (0%–5%)	Slope ratio (0%–5%/0%–100%)	R2 (0%–10%)	WRMSEsd (σ; 0%–10%)	NRMSE (%) (ref = 10%; 0%–10%)	RMSE (0%–10%)	Slope ratio (0%–10%/0%–100%)
**M5**	0.221	3.543	63.101	3.241	1.523	0.651	9.441	43.331	4.332	2.042
**M5+LAMP**	0.225	4.820	24.600	1.333	0.682	0.861	4.606	13.733	1.415	1.048
**DM10**	0.214	6.811	82.601	4.121	2.755	0.604	6.517	55.117	5.529	2.590
**DM10+LAMP**	0.022	11.742	118.202	5.932	3.554	0.482	11.248	167.405	83.681	3.477
**Y119‐RBD7**	R2 (0%–5%)	**WRMSEsd** **(σ; 0%–5%)**	**NRMSE (%)** **(ref = 5%; 0%–5%)**	**RMSE** **(0%–5%)**	**Slope ratio** **(0%–5%/0%–100%)**	R2 (0%–10%)	**WRMSEsd** **(σ; 0%–10%)**	**NRMSE (%)** **(ref = 10%; 0%–10%)**	**RMSE** **(0%–10%)**	**Slope ratio** **(0%–10%/0%–100%)**
**M5**	0.225	28.542	21.908	3.350	2.258	0.617	26.940	26.465	5.370	2.570
**M5+LAMP**	0.455	7.752	21.355	3.633	2.912	0.624	10.895	24.039	5.292	2.547
**DM10**	0.244	41.343	30.878	6.091	3.900	0.505	40.891	30.492	7.539	3.071
**DM10+LAMP**	0.391	14.148	21.925	3.724	2.419	0.639	13.355	21.070	4.632	2.175
**N47‐RBD7+ Y119‐RBD6**	R2 (0%–5%)	**WRMSEsd** **(σ; 0%–5%)**	**NRMSE (%)** **(ref = 5%; 0%–5%)**	**RMSE** **(0%–5%)**	**Slope ratio** **(0%–5%/0%–100%)**	R2 (0%–10%)	**WRMSEsd** **(σ; 0%–10%)**	**NRMSE (%)** **(ref = 10%; 0%–10%)**	**RMSE** **(0%–10%)**	**Slope ratio** **(0%–10%/0%–100%)**
**M5**	0.329	15.408	23.746	6.531	4.258	0.473	14.579	23.281	7.567	2.833
**M5+LAMP**	0.213	23.765	23.566	6.900	4.231	0.456	22.383	26.182	8.975	3.477
**DM10**	0.216	35.245	16.368	4.656	1.781	0.519	33.026	18.578	6.212	2.353
**DM10+LAMP**	0.027	50.282	30.416	10.791	5.996	0.347	47.089	31.498	12.749	4.450

**Table footnote. R^2^**: Coefficient of determination, indicating the proportion of variance in the observed data explained by the fitted model. **RMSE**: Root mean square error, reflecting the average magnitude of deviation between fitted values and observed values. **NRMSE**: Normalized root mean square error, calculated by dividing RMSE by the reference signal at the upper bound of the evaluated interval and expressing the result as a percentage; the reference is the 5% VAF signal for the 0%–5% range and the 10% VAF signal for the 0%–10% range. **WRMSEsd**: RMSE normalized by the experimentally measured standard deviation σ, representing error in units of experimental noise. **Slope ratio**: fitted slope in the low‐VAF interval divided by the fitted slope in the corresponding full range. Higher R^2^ and slope ratio close to 1, and lower RMSE, NRMSE, and WRMSEsd, indicate better low‐frequency quantitative performance.

Amplification is not uniformly beneficial, because its net effect depends on the baseline activity of a given miniCas13d‐guide pair. For unmodified miniCas13d, LAMP markedly improved EP17‐A2 sensitivity: the fLoD decreased from 2.67% to 1.53% with M5 and from 4.11% to 1.16% with DM10 (Figure 7A–C). In parallel, we assessed quantitative behavior specifically within the 0%–10% VAF regime by analyzing this window independently rather than relying on a single full‐range calibration, since broad‐range fits can be disproportionately influenced by the high‐end points and may not reflect low‐end performance. Using the initial reaction slope as the response variable, the NRMSE within 0%–10% VAF decreased from 43.3% to 13.7% for M5 upon LAMP, indicating substantially reduced dispersion around the low‐range trend. We also summarized low‐end gain using a relative low‐range sensitivity ratio, defined as the slope of the dose‐response fit in 0%–10% VAF divided by the slope obtained over 0%–100% VAF. This ratio shifted from 2.04 to 1.05 with M5+LAMP, consistent with a more proportional response at the low end after amplification. Given the potential nonlinearity of Cas13 dose‐response curves, this slope‐based metric was interpreted as a local descriptor of sensitivity rather than evidence of strict global linearity. These results indicate tighter and more well‐behaved low‐end performance with amplification. In contrast, dual‐RBD Cas13d variant already exhibited high intrinsic sensitivity, where amplification elevated background and degraded low‐end behavior. Direct detection with DM10 achieved the lowest fLoD at 0.59%, but LAMP worsened the threshold to 2.30% and increased deviation from global calibration, yielding a slope ratio farther from unity (Figures 7A‐D). NRMSE (0%–10%) increased from 18.6% to 31.5%, and both WRMSEsd and RMSE% rose. For single‐RBD variant, direct detection with DM10 attained an fLoD of about 0.61%. Adding LAMP raised the threshold to roughly 1.66% but improved low range fidelity, reducing WRMSEsd (0%–10%) from about 40.9 to about 13.4 and lowering NRMSE from 30.5% to 21.1%.

Across miniCas13d configurations, a simple pairing rule emerges: DM10 with direct detection most readily achieves the lowest thresholds, whereas M5 with LAMP delivers the most stable low VAF quantitation. On unmodified miniCas13d, M5+LAMP minimized low range dispersion and shifted 0%–10%/0%–100% slope ratio toward unity, with NRMSE reduced to about 13.7%. On single‐RBD variant, it likewise improved quantitative behavior in the 0%–5% window. In contrast, on dual‐RBD variant, amplification raised background and degraded low‐end fidelity, making direct detection with DM10 the preferred setting. The radar plots in Figure 7D illustrated a consistent trend, in which the recommended pairings reach more favorable and more balanced values across the normalized axes while maintaining slope ratios close to one, reflecting the trade‐off between sensitivity and fidelity across backbones.

### 2.7 Detection of Clinical Samples With Engineered miniCas13d‐RBD Variants

**FIGURE 8:**
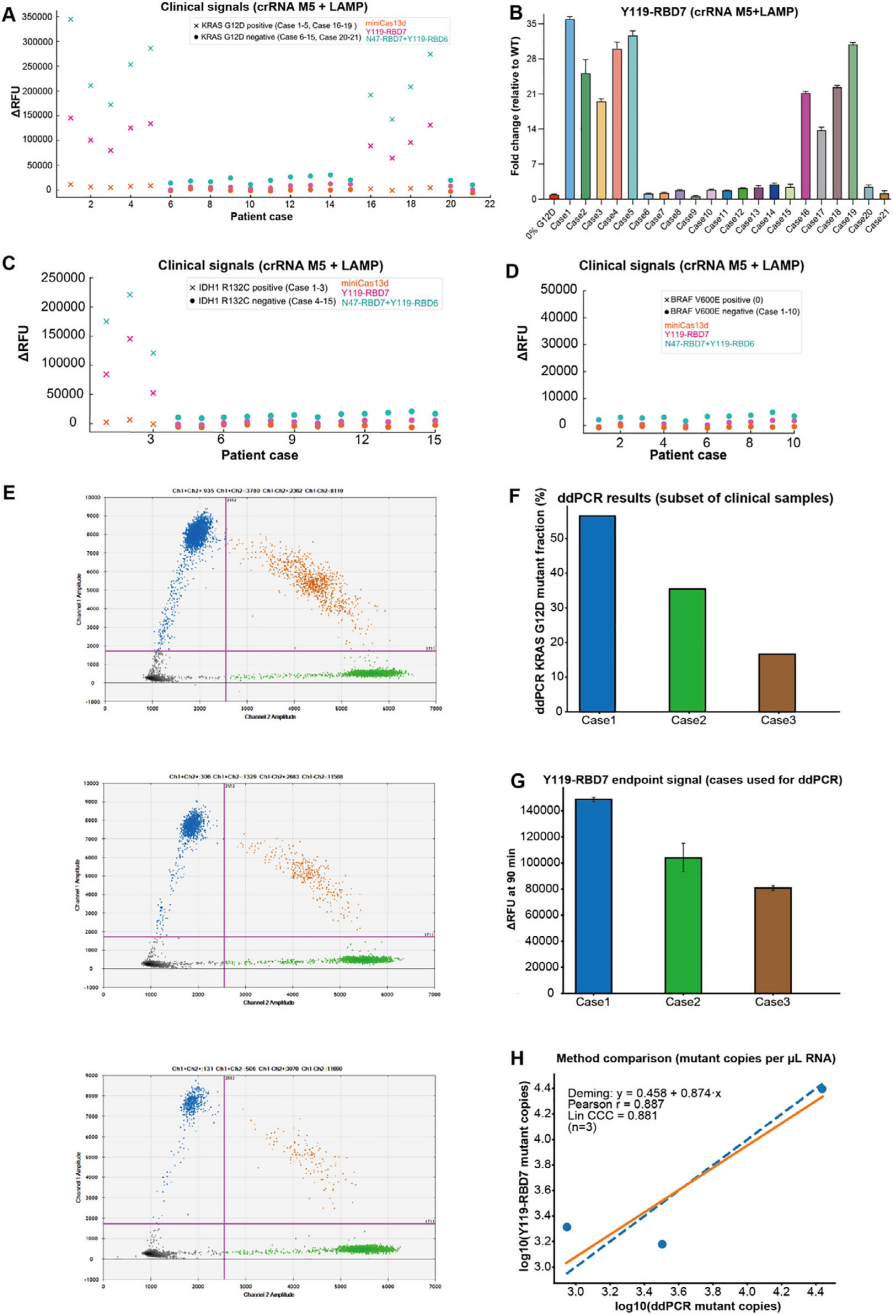
Programmable modular Cas13d assay for clinical sample validation. (A) *KRAS* G12D cohort tested with M5 crRNA and LAMP amplification. Each point represents the mean endpoint fluorescence from triplicate measurements for miniCas13d (orange), Y119‐RBD7 (magenta) and N47‐RBD7+Y119‐RBD6 (cyan). Crosses denote clinically genotyped *KRAS* G12D positive cases. Cases 1–5 and 16–18 were PDAC, whereas case 19 was CRC. Circles denote *KRAS* G12D negative controls (Cases 6–15 and 20–21). (B) Fluorescence fold change at 90 min for Y119‐RBD7 with crRNA M5 and LAMP amplification, normalized to the 0% G12D control. Bars show mean ± SD (n = 3). (C) *IDH1* R132C testing with crRNA M5 and LAMP amplification. Crosses indicate *IDH1* R132C positive cases (cases 1–3), and circles indicate *IDH1* WT controls (cases 4–15). (D) *BRAF* V600E specificity evaluation with crRNA M5 and LAMP amplification in BRAF V600E negative clinical samples (cases 1–10). (E) Representative RT‐ddPCR two‐dimensional amplitude plots for a subset of *KRAS* cases used for orthogonal validation (cases 1–3). (F) ddPCR‐derived *KRAS* G12D mutant fraction for the specimens shown in panel E. (G) Corresponding Y119‐RBD7 endpoint signals (mean ± SD, n = 3) for the same cases. (H) Relationship between Y119‐RBD7 endpoint signal and RT‐ddPCR‐derived mutant burden for the ddPCR‐matched cases shown in panels E‐G. Each point represents one clinical case.

We next evaluated the modular miniCas13d platform in 45 clinical tumor RNA specimens, including 20 pancreatic ductal adenocarcinoma (PDAC), 15 intrahepatic cholangiocarcinoma (ICC), and 10 colorectal cancer (CRC) samples, covering *KRAS* G12D, *IDH1* R132C, and *BRAF* V600E testing contexts (Figure 8A–D). For *KRAS* G12D detection, the analysis set comprised 20 PDAC specimens, including 8 mutation‐positive cases, together with one additional *KRAS* G12D‐positive CRC specimen (Figure 8A). In this set, unmodified miniCas13d provided baseline separation between *KRAS* G12D positive and negative specimens, with the lowest positive exceeding the highest negative by 3.1‐fold (Figure 8A). The single‐RBD variant Y119‐RBD7 expanded dynamic range while maintaining low negative background, increasing positive‐negative separation to 5.5‐fold (Figure 8B), whereas the dual‐RBD variant N47‐RBD7+Y119‐RBD6 produced the highest absolute positive signals but a modestly elevated negative baseline (Figure 8A). Across all three assay variants, all specimens in this *KRAS* G12D detection set were correctly classified in complete concordance with routine clinical genotyping.

We further evaluated *IDH1* R132C in 15 ICC specimens, including 3 mutation‐positive cases, and *BRAF* V600E in 10 CRC specimens, all of which were clinically negative. Across these cohorts, Y119‐RBD7 robustly separated mutation‐positive from mutation‐negative specimens for both *KRAS* G12D and *IDH1* R132C, while maintaining low background across the corresponding negative cohorts (Figure 8B,C). For *BRAF* V600E, signals from the clinically negative CRC cohort remained within the negative range across all cases, indicating no spurious positives in this set (Figure 8D). Together, these expanded cohorts enable assessment of assay robustness across multiple loci and distinct clinical matrices.

To orthogonally validate mutant burden, we analyzed a subset of three *KRAS* G12D‐positive cases by RT‐ddPCR (Figure 8E). RT‐ddPCR quantified *KRAS* G12D mutant fractions spanning 56.8% (Case1), 35.5% (Case2), and 16.5% (Case3) (Figure 8F), which matched the rank ordering of Y119‐RBD7 endpoint signals (Figure 8G). The Y119‐RBD7 endpoint signals were consistent with the RT‐ddPCR‐derived mutant burden across these three cases (Figure 8H).

Finally, we performed a prospective clinical matrix spike‐in to quantify sub‐1% detection for *IDH1* R132C directly in a realistic RNA background without pre‐amplification (Figure S12A–D). Total RNA from *IDH1* R132C negative clinical specimens was used as the matrix, and in vitro‐transcribed mutant *IDH1* R132C RNA was spiked in at 0%, 0.3%, 0.6%, and 1% VAF equivalents with LAMP off. Time‐course measurements showed clear separation of the higher spike‐in levels from the negative matrix (Figure S12A). At the 90‐min endpoint, we defined a decision threshold from the 0% controls, corresponding to 4538.4 ΔRFU (Figure S12B), and used this threshold to classify the measured reactions. No reactions exceeded the cutoff at 0% or 0.3%, whereas all eight replicates exceeded the cutoff at 0.6% and 1% VAF (Figure S12C). Complete detection was observed at 0.6% VAF (Figure S12C,D), supporting sub‐1% detection directly in clinical RNA without pre‐amplification.

## 3 Discussion

**FIGURE 9:**
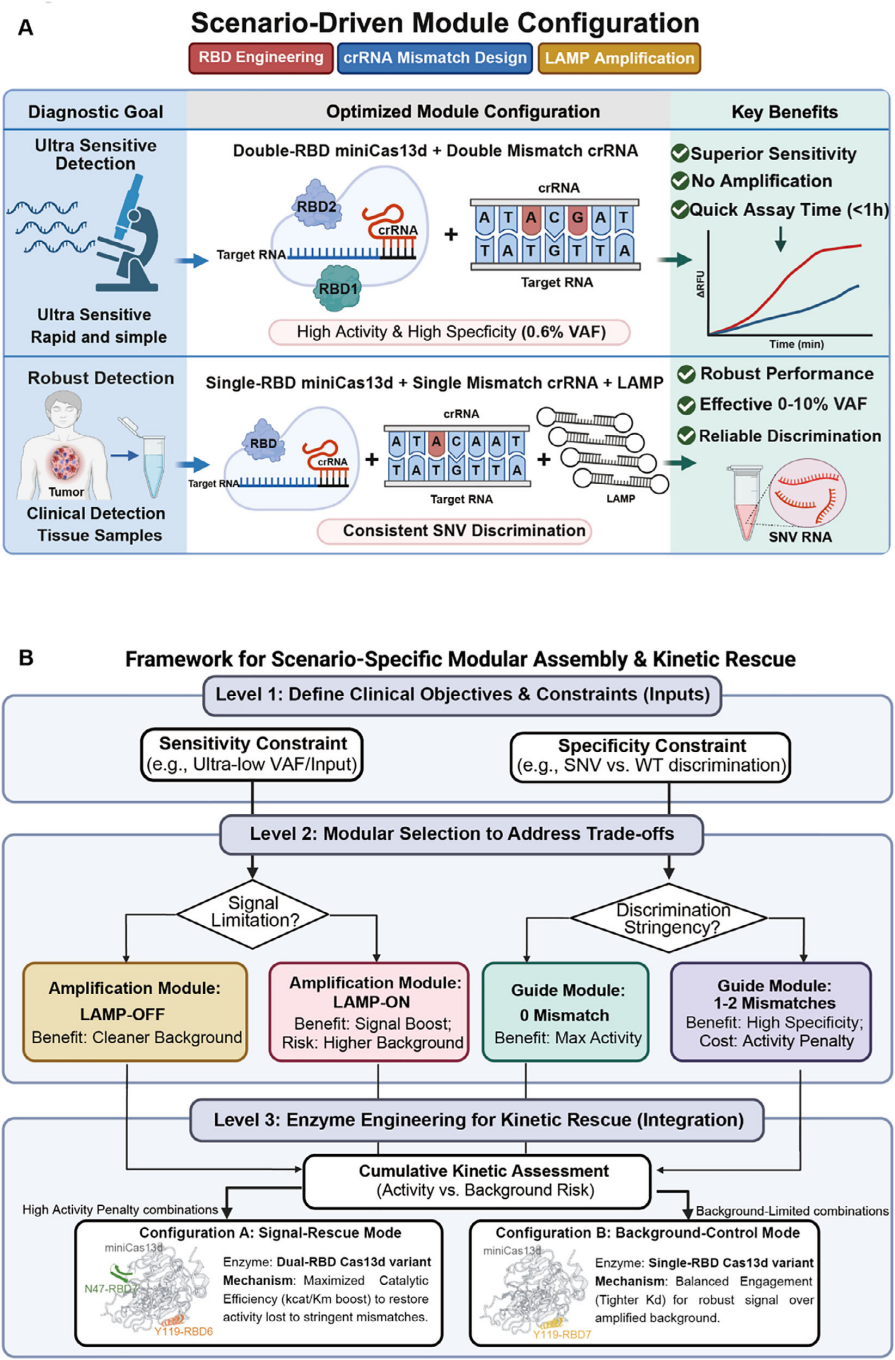
Programmable modular Cas13d platform for RNA SNV detection. (A) Schematic overview of the three interchangeable assay modules, including RBD engineering, crRNA mismatch design, and optional LAMP amplification. Two representative scenario‐driven configurations are shown. For ultra‐sensitive, amplification‐free detection, a double‐RBD miniCas13d is paired with a double‐mismatch crRNA to achieve high activity and high specificity at low VAF. For robust detection in clinical tissue samples, a single‐RBD miniCas13d is combined with a single‐mismatch crRNA and LAMP amplification to improve signal robustness and discrimination across the 0%–10% VAF range. (B) Scenario‐specific decision framework for modular Cas13d RNA SNV detection. Clinical deployment begins by defining the dominant constraint, such as ultra‐low input sensitivity or stringent SNV‐versus‐WT discrimination. Modules are then selected to address the major trade‐off: LAMP‐OFF minimizes background, whereas LAMP‐ON boosts signal at the cost of higher background. Guide design can be set to 0 mismatch for maximal activity or to 1–2 mismatches for greater specificity with reduced activity. Enzyme engineering is then used for kinetic rescue and module integration. Configuration A uses a dual‐RBD Cas13d variant to restore activity under stringent mismatch conditions, whereas Configuration B uses a single‐RBD Cas13d variant to improve target engagement and maintain robustness under background‐limited settings.

This study addresses a central constraint in tumor‐associated RNA SNV detection: mismatch‐engineered crRNAs improve allelic discrimination by suppressing WT activation, but often impose a substantial activity penalty that becomes limiting at low input or low VAF [39, 43, 44, 45]. We approached this constraint as an engineering problem with separable levers and organized assay design into a three‐module Cas13d framework—crRNA mismatch, protein engineering, and optional isothermal amplification—that can be tuned independently and recombined to meet a predefined clinical objective (Figure 9A,B). In addition, we considered stoichiometric tuning as an assay‐level operating condition that complements these modular levers rather than a separate module [46, 47]. In practice, the Cas13d:crRNA assembly ratio and reporter load define the operating point at which any crRNA, engineered protein, or amplification module is evaluated. Based on systematic optimization, we standardized benchmarking at 50 nM protein with a 1:1 protein:crRNA ratio, which maximized usable dynamic range while maintaining low baseline.

At the crRNA level, scanning mismatch positions across multiple oncology hotspots revealed two reproducible discrimination windows (M2‐M6 and M17‐M22) that consistently preserved mutant signal while strongly suppressing WT activation. Structural mapping of these windows onto a miniCas13d‐crRNA‐target RNA ternary model is consistent with a two‐checkpoint interpretation [18, 30]: a distal window that primarily perturbs early engagement and productive binding, and a proximal window near the crRNA handle and HEPN region that preferentially disrupts activation gating. This framing makes mismatch design more portable and predictable by linking mismatch placement to whether the desired penalty is exerted at binding versus activation.

At the engineered‐protein level, structure‐guided internal insertion of auxiliary RNA binding domains provided kinetic rescue of mismatch‐imposed activity loss without erasing the positional specificity rules. The N47 and Y119 insertion sites were selected for structural permissiveness and functional proximity to the crRNA handle region or HEPN lobe [29, 31, 32, 35, 48]. Consistent with this design, engineered variants preserved mismatch sensitivity patterns while increasing output. Kinetic measurements further indicated that RBD fusions improve performance through coupled effects on engagement and catalysis, reflected by reduced apparent activator‐binding *K*_d_ and improved catalytic efficiency. Notably, the dual‐RBD variant N47‐RBD7+Y119‐RBD6 provided an additional gain over either single insertion, most clearly manifested as higher *k*_cat_ together with lower *K*_m_, consistent with partially additive functional improvements rather than indiscriminate signal inflation.

Amplification is treated as an optional lever whose benefit is module‐dependent [36, 49, 50]. In low‐activity contexts, LAMP increased effective template availability and improved fractional detection limits for unmodified miniCas13d. In high‐activity Cas13d variants or stringent double‐mismatch guides, amplification did not necessarily improve the low‐VAF decision boundary and could worsen it by elevating background. Accordingly, amplification cannot be assumed beneficial and must be evaluated jointly with mismatch crRNA and engineered protein variants.

To compare these trade‐offs in the regime that matters clinically, we adopted CLSI EP17‐A2 decision limits (LoB and LoD) using the initial reaction slope as the primary quantitative readout and explicitly analyzed 0%–10% VAF window with normalized low‐range error metrics. This framework translated to clinical testing: engineered scaffolds increased signal range in patient RNA while maintaining the expected trade‐off between activity and baseline. The single‐RBD variant Y119‐RBD7 improved positive‐negative separation in a *KRAS* G12D cohort, whereas the dual‐RBD variant maximized absolute signal but could raise baseline in some settings, motivating objective‐dependent configuration choice. Beyond *KRAS*, we observed full concordance for *IDH1* R132C and *BRAF* V600E clinical specimens, and a prospective clinical‐matrix spike‐in for *IDH1* R132C showed complete detection at 0.6% VAF without pre‐amplification, supporting rule‐guided module selection toward a predefined sub‐1% objective [51, 52].

Several limitations define the current boundaries. First, RNA abundance and integrity vary across specimen types, collection protocols, and tumor contexts. Variant allele fraction at RNA level is influenced by allele‐specific expression, transcript stability, and tumor cellularity [53, 54, 55, 56]. These factors can decouple RNA readouts from DNA allele fractions and can shift optimal configuration choices. Second, background behavior under amplification depends on primer design, matrix composition, and guide architecture. In high activity Cas13d variants, amplification can raise background enough to offset gains in signal [50]. Third, the clinical cohort is a pilot‐scale study. Larger cohorts with quantitative reference standards are needed to establish performance distributions, to evaluate interlaboratory reproducibility, and to define clinically meaningful cutoffs for specific use cases. Fourth, the current work focuses on a limited set of hotspots and guide architectures. Although mismatch windows were conserved across the tested targets, generalization to broader variant classes should be evaluated systematically.

In summary, this work frames RNA SNV detection by Cas13d as a configurable system rather than a single fixed pipeline. The central specificity‐sensitivity tension is addressed by three linked programmable modules. crRNA mismatch placement is made rule‐based and mechanistically interpretable through two checkpoint windows. Activity loss is compensated by structure‐guided protein engineering on a compact miniCas13d scaffold using modular RNA binding domains. Amplification is treated as a conditional module whose benefit is quantified using EP17‐A2 decision limits and low VAF error analysis. The resulting framework supports selection of operating modes that align with clinical objectives and provides a path toward practical CRISPR‐based SNV diagnostics across diverse oncology targets.

## 4 Methods

### 4.1 Plasmid Construction

Coding sequences for selected RBDs, including U1A (SNRPA, UniProt P09012, RefSeq NM_004596), HuR (ELAV1, UniProt Q15717, RefSeq NM_001419.3), hnRNPK (HNRPK, UniProt P61978, RefSeq NM_001318186.2), and ZFP36 (TTP, UniProt P22893, RefSeq NM_003407.5), were retrieved from UniProt and GenBank. Gene specific primers were designed using NCBI Primer BLAST and analyzed with OligoAnalyzer 3.1 to ensure specificity and optimal melting temperatures. Each primer pair was also checked by BLAST against human genome (GRCh38.p14) to confirm unique targeting. cDNA from SW1990 cells was used as template for PCR amplification of each RBD coding region. High fidelity PCR products were confirmed by agarose gel electrophoresis and purified.

RBD fragments were inserted into a modified pET‐28b miniCas13d expression vector (N‐terminal 6×His‐SUMO tag) using two complementary methods. For overlap extension PCR, overlapping fragments (20–30 bp) were amplified and fused to generate full‐length inserts with vector‐compatible homology arms, which were purified and cloned. For homologous recombination, vectors were linearized by restriction digestion or inverse PCR, and assembled with purified inserts using ClonExpress II (Vazyme, China, C112) at 37°C for 30 min. Recombinant plasmids were transformed into chemically competent *E. coli* DH5α (WEIDI Bio, China, DB106), screened by colony PCR, and confirmed by Sanger sequencing to ensure correct orientation and in‐frame fusion. Verified plasmids were used for downstream assays.

### 4.2 Protein Expression and Purification

The validated miniCas13d and RBD‐fusion plasmids (modified pET‐28b with an N‐terminal 6×His‐SUMO tag) were transformed into *E. coli* Rosetta 2 (DE3) cells (WEIDI bio, China, EC1002). After overnight growth at 37°C, the culture was expanded in 2×TY medium and induced with 0.2 mM isopropyl β‐D‐1‐thiogalactopyranoside (IPTG) at 16°C for 16–18 h. Cells were harvested by centrifugation and resuspended in lysis buffer (50 mM Tris, pH 8.0, 800 mM NaCl, 10% glycerol) supplemented with protease inhibitor cocktail. After high pressure homogenization followed by sonication, the lysate was clarified by centrifugation to remove cell debris.

Recombinant proteins were purified by Ni‐NTA affinity chromatography, followed by on‐column SUMO tag cleavage using ULP1 protease. The cleaved proteins were further purified by size exclusion chromatography on a Superdex 200 Increase 10/300 GL column equilibrated in 50 mM Tris‐HCl pH 8.0, 150 mM NaCl, using an ÄKTA Pure system (Cytiva, United States). Protein purity and molecular weight were verified by SDS‐PAGE using molecular weight standards. The A260/A280 ratio was used to confirm minimal nucleic acid contamination. Protein concentrations were determined by BCA assay (Beyotime, China, P0012) or by absorbance at 280 nm using calculated extinction coefficients. Purified proteins were concentrated, aliquoted, snap frozen in liquid nitrogen, and stored at −80°C.

### 4.3 Generation of Target RNAs

Reference coding sequences for human *KRAS* (NM_033360.4), *BRAF* (NM_004333.6), and *IDH1* (NM_005896.4) were obtained from NCBI GenBank. Oncogenic hotspot mutations *KRAS* G12D (c.35G>A), *BRAF* V600E (c.1799T>A), and *IDH1* R132C (c.394C>T) were identified using the COSMIC database. Primers were designed with Primer Premier 6.0 to amplify DNA fragments encompassing the mutation sites. A T7 promoter sequence was appended to the 5′ end of the forward primers, and reverse primers were selected to minimize secondary structures. Primer properties, including melting temperature (Tm), GC content, and secondary structures, were validated using OligoAnalyzer 3.1.

Total RNA was extracted from SW1990, Pan1005, and other relevant cell lines using TRIzol reagent following manufacturer's protocol. First‐strand cDNA synthesis was performed using HiScript III first Strand cDNA Synthesis Kit (+ gDNA wiper) (Vazyme Biotech Co., Ltd., China) to eliminate genomic DNA contamination and ensure high fidelity reverse transcription. The resulting cDNA was used as a template for amplifying *KRAS* (from SW1990), *IDH1* (from SW1990), and *BRAF* (from Pan1005) coding regions. PCR amplification was performed using Phanta Flash Master Mix (Vazyme Biotech Co., Ltd., China, P520) with target specific primers under optimized annealing temperatures. PCR products were analyzed by agarose gel electrophoresis, and the expected bands were excised and purified using TIANGEN Gel Purification Kit (TIANGEN, China, DP209‐03).

Purified DNA fragments were transcribed in vitro using T7 High Yield Transcription Kit (Thermo Scientific, United States, K0441), and RNA products were purified with TRIzol reagent following the manufacturer's instructions. Then RNA was aliquoted and stored at −80°C for long‐term preservation.

### 4.4 Design and Optimization of Cas13d SNV Specific crRNAs

**In silico selection**. Mutant transcript sequences corresponding to *KRAS* G12D, *IDH1* R132C, and *BRAF* V600E were subjected to guide RNAs (gRNAs) design using the publicly available Cas13design platform (http://cas13design.nygenome.org) [39]. Candidate gRNAs were ranked by predicted efficacy (TIGER score), and the top ten for each target were retained. To evaluate SNV specificity, the corresponding wild type sequences were also analyzed, and predicted activities of wild type targeting guides (Score_WT) were obtained. A specificity metric (ΔScore = Score_SNV − Score_WT) was used, with ΔScore ≥ 0.2 considered discriminatory. All selected guides satisfied this threshold.

**crRNA preparation**. The selected 23‐nt guide sequences were converted into full crRNAs by appending the invariant direct repeat sequence. DNA templates for crRNAs were generated by overlap extension PCR, in which forward primers carried a T7 promoter at the 5′ end and reverse primers annealed to the 3′ end of direct repeat. PCR products were gel‐purified and used as templates for in vitro transcription with T7 High Yield Transcription Kit. Transcribed crRNAs were purified with TRIzol, quantified by NanoDrop, and stored at −80°C.

**Rational mismatch engineering**. To further improve SNV discrimination, systematic mismatch libraries were constructed for each target. single mismatch libraries were generated by substituting one nucleotide at each of 23 spacer positions, yielding 23 variants per target. Each guide was expected to introduce a single mismatch with mutant RNA but two mismatches with wild type sequence, thereby enhancing allelic specificity.

Building on these designs, double mismatch libraries were created by introducing paired substitutions at 12 selected spacer position combinations per target. These guides generated two mismatches with mutant RNA and three with wild type sequence, providing an additional layer of discrimination. Collectively, these engineered crRNA libraries enabled a systematic evaluation of how deliberate mismatches modulate the selectivity of Cas13d for oncogenic SNVs.

### 4.5 Design and Optimization of LwaCas13a SNV Specific crRNAs

LwaCas13a crRNAs for SNV detection were designed using two approaches. crRNA1 was designed according to the SHERLOCK synthetic mismatch principle, in which the variant nucleotide is preferably positioned at position 3 of the 5’ seed region to maximize allelic discrimination. For *KRAS* G12D target, placement at position 3 was not feasible because of protospacer flanking site constraints; the variant was therefore positioned at position 4. An additional synthetic mismatch was introduced at position 5, generating the optimized crRNA1 M5, which was used in subsequent experiments.

crRNA2 was generated by adapting the Cas13d guide sequence used in this study to the LwaCas13a crRNA architecture. Because the Cas13d‐derived spacer was 4 nucleotides shorter than the spacer length used for the optimized LwaCas13a guide RNA, namely crRNA1, a 4‐nucleotide extension was added to the 3’ end of the spacer to match guide length. This extension was introduced solely to satisfy the spacer‐length requirement of LwaCas13a and was independent of the synthetic mismatch design.

### 4.6 Fluorescent Reporter Cleavage Assay

Stoichiometry optimization using the M0 guide was performed to determine the optimal molar ratio of miniCas13d to its cognate crRNA. MiniCas13d and the cognate crRNA were pre‐assembled at molar ratios of 1:1, 2:1, and 1:2. For each tested condition, the final protein concentration was set to 10, 50, or 100 nM, and crRNA concentration was adjusted accordingly to achieve the indicated molar ratio. Ribonucleoprotein complexes were prepared at double the final working concentration, and 5 µL was mixed with 5 µL of the remaining reaction components to yield a final reaction volume of 10 µL. The optimized condition, composed of 50 nM miniCas13d and 50 nM crRNA at a 1:1 molar ratio, was adopted for all subsequent experiments.

Unless otherwise indicated, cleavage reactions (10 µL) contained 50 nM miniCas13d, 50 nM crRNA, 50 nM target RNA, and 50 nM reporter (5’‐FAM‐10U‐BHQ1‐3’) in cleavage buffer (50 mM Tris‐HCl, pH 8.0; 150 mM NaCl). MiniCas13d and crRNA were pre‐incubated at room temperature for 10 min prior to addition of target RNA and reporter. Reactions were briefly centrifuged (5000 rpm, 5 s) and then incubated at room temperature for measurement.

Real time fluorescence was measured on a BioTek Synergy H1 plate reader with excitation at 488 nm and emission at 520 nm. Fluorescence signals were recorded every 15 min for 90 min and expressed as relative fluorescence units (RFU). The 15‐min value was used as the baseline to minimize startup fluctuations in the initial readout. Fluorescence change was calculated as ΔRFU(t) = RFU(t)−RFU(t_15min_). All reactions were performed in three independent replicates, and results are reported as mean ± SD. Statistical significance was assessed using two‐tailed independent samples t‐tests in GraphPad Prism 9.0.

### 4.7 In Silico Design of miniCas13d‐RBD Variants

Ten RBDs were selected based on sequence annotation and functional characterization, including canonical modules such as RNA recognition motifs (RRM), K‐homology (KH) domains, and zinc finger (ZF) motifs. Each RBD was inserted at four candidate positions within miniCas13d, yielding 40 fusion variants. Structural predictions were performed with AlphaFold3 [41]. For each variant, five independent structural models were generated, and only those with high per‐residue confidence (pLDDT > 70) and favorable interfacial scores (ipTM > 0.55) were retained.

To evaluate structural impact of RBD insertions on RNA recognition, ternary complexes comprising miniCas13d, crRNA, and target RNA were modeled for both wild type and engineered fusions. Predicted complexes were visualized in PyMOL 2.5 to compare RNA binding orientations and interfaces in the presence or absence of fused RBD. From these analyses, eight fusion designs were identified as top candidates, preserving overall miniCas13d structural integrity while introducing additional potential RNA contacts. These candidates were prioritized for subsequent experimental validation.

### 4.8 Measurement of Binding Affinity (K_d_)

Purified Cas13 variants were complexed with the corresponding crRNA to form RNPs and equilibrated at room temperature in binding buffer (50 mM Tris‐HCl, pH 8.0; 150 mM NaCl). To prevent probe cleavage, a nuclease‐resistant, fluorescently labeled target RNA (fully 2’‐O‐methyl; “*” denotes phosphorothioate linkages) was used: FAM‐G*A*UGGCGUAGGCAAGAGUGCC*U*U. Binding affinity to the target RNA was quantified by fluorescence polarization. A fixed concentration of 1 nM fluorescent probe was incubated with a titration series of RNPs spanning 0.1–100 nM, and fluorescence polarization (mP) was recorded after equilibration. Binding data were fit by nonlinear regression to a single site binding isotherm, and fitted parameters are reported with 95% confidence intervals.

### 4.9 Steady‐State Kinetics of Cas13‐mediated Collateral RNA Cleavage

To ensure stable activation while measuring collateral kinetics, 1 nM RNPs were pre‐activated with an unlabeled, nuclease‐resistant activator G*A*UGGCGUAGGCAAGAGUGCC*U*U (fully 2’‐O‐methyl; “*” denotes phosphorothioate linkages). Reactions were initiated by adding a quenched fluorogenic RNA reporter across a concentration series of 0–60 nM, and fluorescence was monitored in real time. Fluorescence was converted to product concentration using a calibration curve, and initial rates *v_0_* were obtained by linear regression over the early linear regime and expressed as initial velocity (nM/min). Apparent *K*_m_ and *V*_max_ were estimated by fitting initial velocity versus reporter concentration to the Michaelis‐Menten model *v_0_* = *V*_max_ [S] / (*K*_m_ + [S]) with *K*_m_, *V*_max_ ≥ 0. Turnover was calculated as *k_cat_* = *V*_max_ / [E]_active,_ where [E]_active_ was defined as the concentration of pre‐activated Cas13 RNP in the reaction, assuming quantitative activation under these conditions. Catalytic efficiency was calculated as *k*_cat_/*K*_m_. 95% confidence intervals were derived from fit uncertainty.

### 4.10 Analytical Sensitivity Evaluation

Analytical sensitivity of miniCas13d and engineered miniCas13d‐RBD proteins for SNV detection was assessed by an in vitro competitive cleavage assay under CLSI EP17‐A2 guidelines [40, 42]. Defined mixtures of SNV and WT RNAs were prepared at allele frequencies of 100%, 99%, 95%, 90%, 75%, 50%, 25%, 10%, 5%, 1%, 0.5%, 0.25%, 0.1%, 0.01%, and 0%. Each reaction contained 50 nM target RNA, 50 nM Cas13d protein, and 50 nM crRNA in cleavage buffer (50 mM Tris‐HCl, pH 8.0, and 150 mM NaCl). Real time fluorescence was measured as described in the reporter cleavage assay. For benchmarking with nucleic acid amplification, LAMP was carried out using a commercial kit (Vazyme, China, RP712) according to manufacturer's instructions. Amplicons were validated by agarose gel electrophoresis, purified, transcribed in vitro, and the RNA products were assayed under identical Cas13d conditions. All reactions were performed in triplicate and results are reported as mean ± SD.

Fluorescence traces were analyzed using a standard pipeline. Signals were baseline corrected by subtracting fluorescence at 15 min, and quantification was restricted to 15–90 min linear response window. Reaction rates were determined as the slope of a linear regression fitted by ordinary least squares across this interval, with outliers removed using a predefined 3.5× median absolute deviation criterion. No‐template controls on each plate provided the analytical background, yielding the mean slope of blanks (μ_blank) and its standard deviation (σ_blank). Analytical thresholds were calculated following CLSI EP17‐A2 guidelines [42]:

The limit of blank (LoB) in slope units was calculated as:

LoB_slope=μ_Blank+1.645×σ_Blank

The limit of Detection (LoD) in slope units was calculated as:

LoD_slope=LoB_slope+1.645×σ_Low

where σ_low represents the standard deviation of slopes at the lowest non‐zero target concentration measured in independent replicates.

Thresholds in concentration units were derived from within system calibration using wild type dilution series run on the same plates. Net slope was calculated as:

Δslope=Slope−μ_Blank

Δslope was regressed against target concentration C with zero intercept to obtain the sensitivity coefficient: b̂ (RFU·min^−1^ per concentration unit)

The limit of detection in concentration space was then calculated as:

$$\mathrm{LoD}\_\mathrm{SNV}=\left(\mathrm{LoD}\_\mathrm{slope}-\mu \_\mathrm{blank}\right)/\hat{b}$$

Relative sensitivity for SNVs was expressed as the fractional LoD:

$$\mathrm{fLoD}=\mathrm{LoD}\_\mathrm{SNV}/C\_\mathrm{ref}\left(100\%\mathrm{SNV}\right)$$

These calculations were performed separately within LwaCas13a and each Cas13d amplification system to avoid cross system confounding.

Uncertainty was estimated by non‐parametric bootstrap and Monte Carlo propagation. At least 2000 bootstrap resamples were generated for μ_blank, σ_blank, and σ_low using well level resampling, and for b̂ using resampling of calibration pairs [C, Δslope]. For each of at least 10 000 Monte Carlo draws, parameters were sampled from the bootstrap distributions and propagated to LoB_slope, LoD_slope, LoD_conc, and fLoD%. Two‐sided 95% confidence intervals were then obtained from the 2.fifth to 97.fifth percentiles, with bias correction and acceleration applied where appropriate. Acquisition settings and the linear analysis window were kept constant across all conditions, ensuring that observed differences in Blank_mean_slope, Blank_SD_slope, LoB_slope, LoD_slope, b̂, and fLoD% reflected true biochemical performance rather than analytical artifacts.

### 4.11 RNA Extraction From Human Tumor Tissue

Total RNA was extracted from liquid nitrogen preserved human tumor tissues using TRIzol reagent (Thermo Fisher Scientific, United States, 15596026). Approximately 30 mg of frozen tissue was pulverized, homogenized in TRIzol, and processed by chloroform extraction, isopropanol precipitation, and 75% ethanol washes. RNA pellets were dissolved in RNase‐free water and quantified by NanoDrop.

### 4.12 Droplet Digital PCR for KRAS G12D Quantification

Droplet digital PCR was performed to quantify *KRAS* c.35G>A (p.G12D) (NM_004985.5) using primers/probes designed in Primer Premier 5.0 (82‐bp amplicon):

Kras G12D‐F, 5′‐Aggcctgctgaaaatgactg‐3′;

Kras G12D‐R, 5′‐Tagctgtatcgtcaaggcactc‐3′;

WT Probe (VIC‐MGB), 5′‐VIC‐TACGCCACCAGCTC‐MGB‐3′;

*KRAS* G12D probe (FAM‐MGB), 5′‐FAM‐TACGCCATCAGCTC‐MGB‐3′.

RNA was reverse‐transcribed to complementary DNA, which was used as input for digital droplet PCR. Reactions were prepared with a total volume of 20 µL. Each reaction contained 10 µL ddPCR Supermix for Probes without dUTP from Bio‐Rad, 1.8 µL of each primer at a concentration of 10 µM, 0.5 µL of each probe at a concentration of 10 µM, 4 µL of template, and 1.4 µL of nuclease‐free water. Droplets were generated using DG8 cartridges and QX200 droplet generation oil from Bio‐Rad on a QX200 Droplet Generator. Generated droplets were transferred to a 96‐well plate, heat‐sealed, and subjected to thermocycling. Thermocycling conditions were set as follows: initial denaturation at 95°C for 10 min, followed by 40 cycles of denaturation at 94°C for 30 s and annealing/extension at 60°C for 60 s, then a final extension at 98°C for 10 min, and a holding step at 4°C. Droplets were read on a QX200 Droplet Reader. Data analysis was performed using QuantaSoft or QuantaSoft Analysis Pro software. Thresholds were set based on control samples to classify FAM‐positive droplets as *KRAS* G12D mutant and VIC‐positive droplets as wild type.

### 4.13 Prospective Clinical RNA Background Spike‐in for Sub‐1% VAF Quantification

Prospective clinical matrix spike‐in validation was performed for *IDH1* R132C. Total RNA (50 ng per 10 µL reaction) from *IDH1* R132C negative clinical specimens served as background matrix. In vitro transcribed *IDH1* R132C mutant transcripts were spiked in at 0%, 0.3%, 0.6%, and 1% VAF equivalents. All reactions were run without pre‐amplification (LAMP OFF). The limit of blank (LoB) was determined using 0% spike‐in controls (n = 8). LoD_95_/fLoD was defined as the lowest VAF with ≥95% of replicate signals above LoB.

## Author Contributions

Zeyu Wang performed all experiments, conducted statistical analyses, and led data visualization and interpretation of results. Jiahao Li provided critical reagents, including cell pellet samples. Zhuying Yue and Qing Xia contributed tumor tissue specimens and managed institutional ethics approvals. Zhen Fang, Beixuan He and Wenhua Zhang contributed to the study design and scientific discussions. Yingbin Liu and Yanjing Li supervised the project, provided conceptual leadership, and co‐led data analysis and manuscript preparation. Zeyu Wang and Yanjing Li prepared the figures and wrote the manuscript.

## Ethics Approval Statement

This study, which involved human pancreatic ductal adenocarcinoma (PDAC), intrahepatic cholangiocarcinoma (ICC), and colorectal cancer (CRC) tumor specimens, was approved by the Institutional Review Board of Renji Hospital Affiliated to Shanghai Jiao Tong University school of Medicine (Approval No.: KY2025‐022‐B, KY2025‐166‐B). Written informed consent was obtained from all participants, and all methods were carried out in accordance with relevant institutional guidelines and the Declaration of Helsinki.

## Conflicts of Interest

The authors declare no competing interests.

## Supporting information




**Supporting File**: advs75015‐sup‐0001‐SuppMat.docx.

## Data Availability

All data needed to evaluate the conclusions of this study are present in the article and its Supplementary Information. Additional data or materials related to this work are available from the corresponding author upon reasonable request.
