# Supplementary material for: A Modular and Programmable Cas13d Platform for RNA Single Nucleotide Variant Detection
Source: Adv Sci (Weinh). 2026 May 4;13(32):e23680. doi: 10.1002/advs.202523680 (PMC13252642; doi:10.1002/advs.202523680)
Supplement: Supplementary file 1 — Supporting File: advs75015‐sup‐0001‐SuppMat.docx. [file ADVS-13-e23680-s001.docx]

**
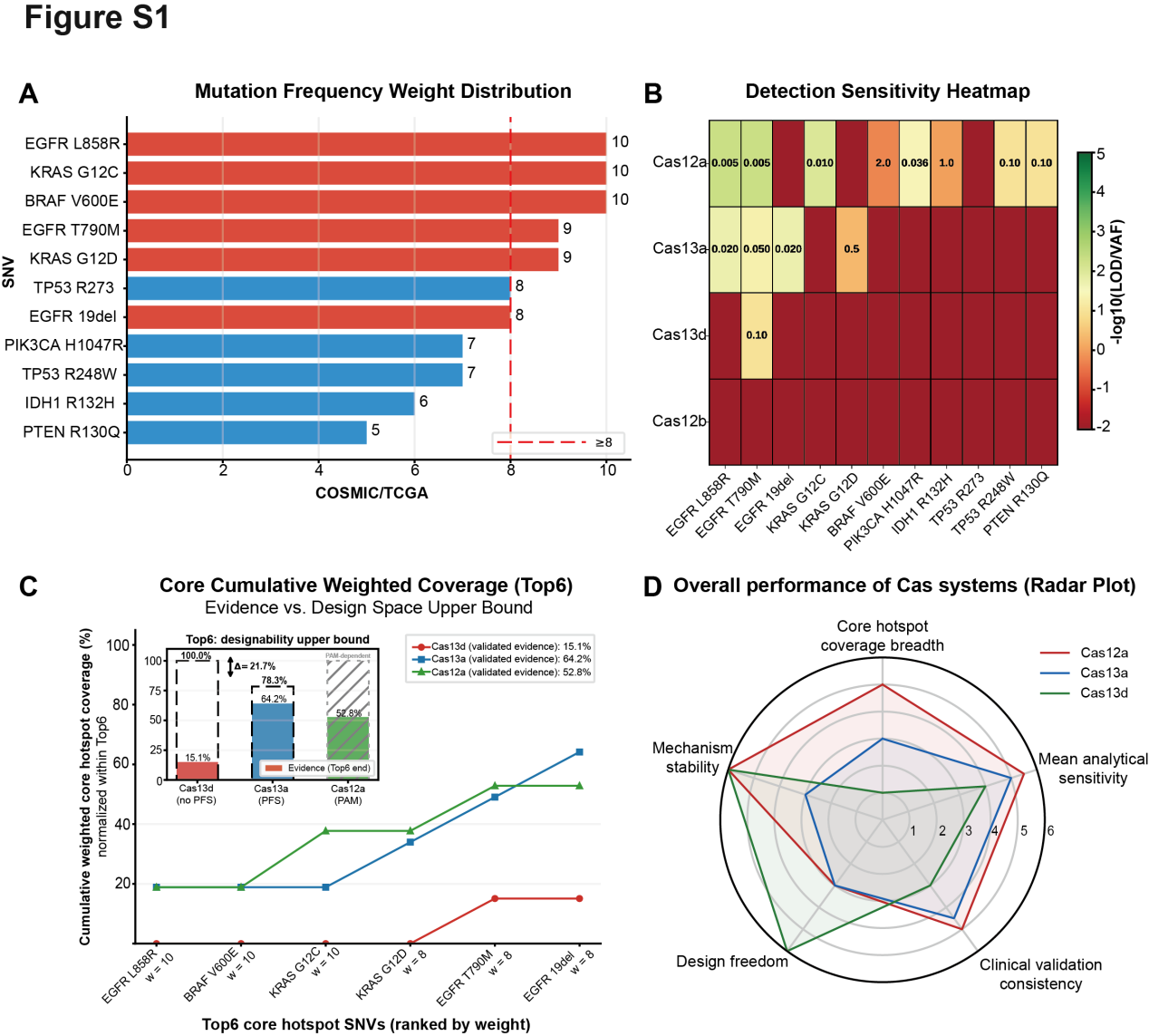
**

**Figure S1. Targetability and analytical performance of Cas systems across high frequency tumor hotspot SNVs**

1. COSMIC/TCGA-derived frequency weights for 11 recurrent hotspot SNVs. The dashed line marks core hotspots with a frequency weight ≥8.

**B.** Detection sensitivity heatmap for Cas12a, Cas13a, Cas13d, and Cas12b across hotspots, shown as -log_10_(LOD/VAF) with LOD (% VAF) annotated. Dark red cells indicate no reported literature evidence.

**C.** Cumulative frequency weighted targetable coverage across the Top6 hotspots, comparing Cas13d, Cas13a and Cas12a. Targetability was defined as an SNV positioned within the crRNA single base discrimination window and detected with an LOD of ≤1% VAF. Dashed bars denote the theoretical upper bound of the design space, whereas the colored bars and line traces represent currently validated evidence.

**D.** Radar summary on a 0-6 scale integrating coverage breadth, mean sensitivity, clinical consistency, design freedom, and mechanistic stability, illustrating the trade-offs between sequence-constrained systems and PFS-free Cas13d for low-VAF SNV detection.

**
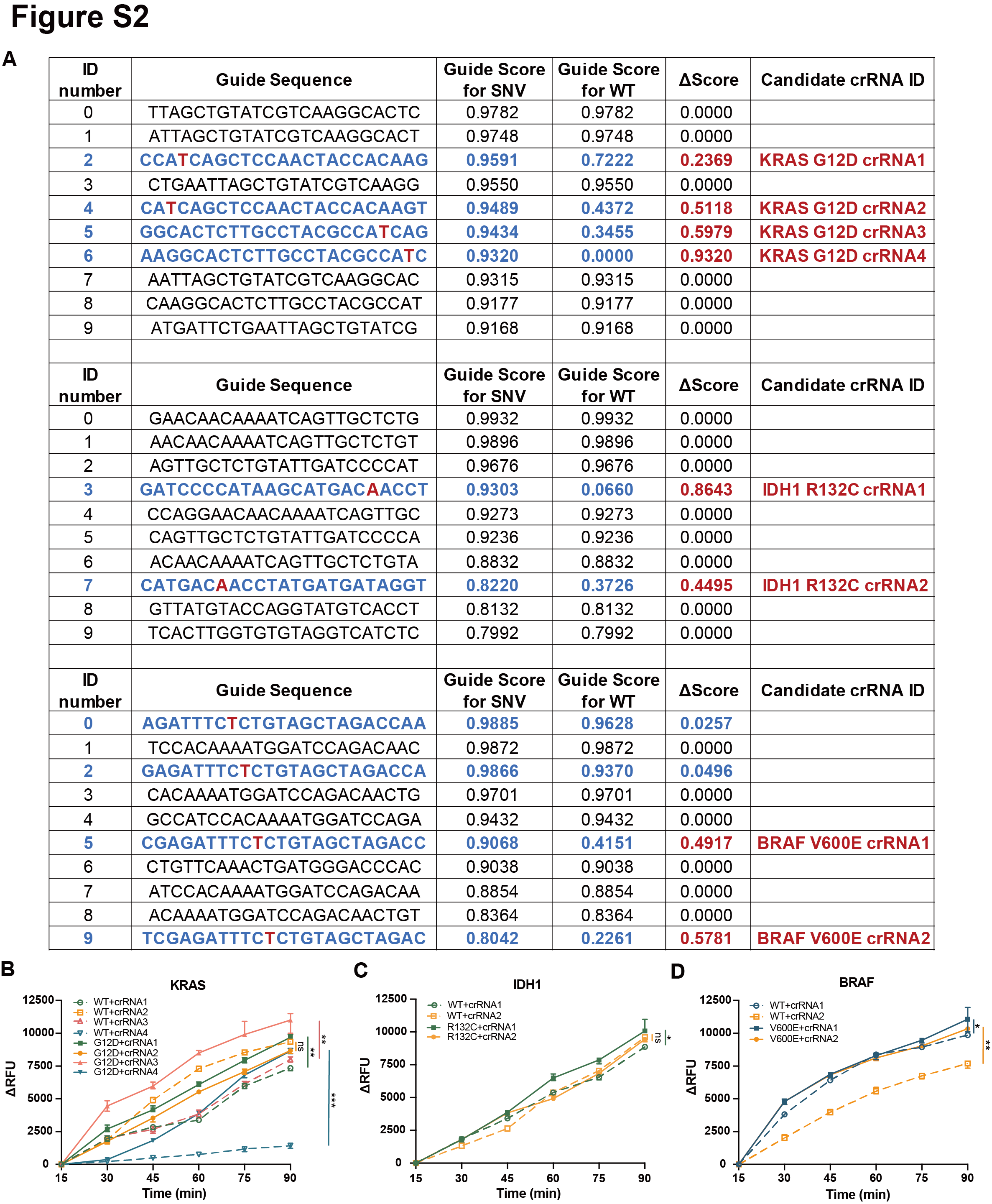
**

**Figure S2. Predicted activity scores and time-course analysis of candidate crRNAs**

**A.** Candidate crRNAs were designed using the Cas13design tool, based on an 80-nt input sequence centered around the SNV site. ΔScore (Score_SNV – Score_WT) was used to prioritize crRNAs for further testing, as described in Figure 1. SNV nucleotides are highlighted in red. crRNAs whose targeting regions include the SNV site are shown in blue; others are in black.

**B-D.** Time course fluorescence cleavage assays comparing WT (dashed lines) and SNV containing (solid lines) RNA targets for *KRAS* (B), *IDH1* (C), and *BRAF* (D). ΔRFU at each time point was calculated as RFU(t) − RFU(t_15min_). Data represent mean ± SD (n = 3). Statistical analysis was performed at 90 min using independent samples t-tests. *p < 0.05; **p < 0.01; ***p < 0.001; ns: not significant.


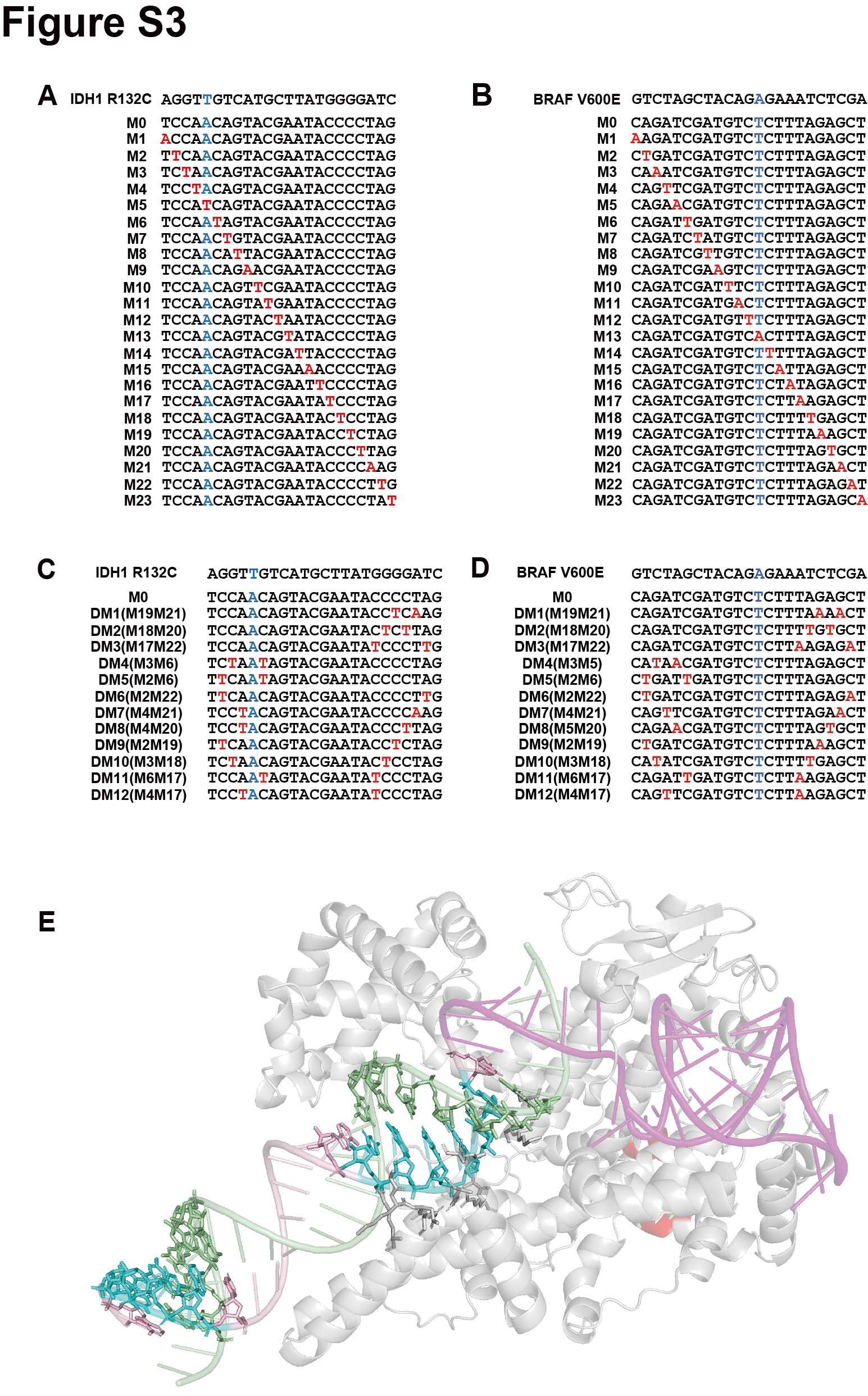


**Figure S3. Systematic mismatch crRNA design for *IDH1* and *BRAF* reveals two mechanistic mismatch windows in miniCas13d.**

**A, B.** Single mismatch crRNAs (M1-M23) were generated by introducing single nucleotide substitutions across crRNA spacer of *IDH1* crRNA1 (A) and *BRAF* crRNA2 (B). Additional engineered mismatches are shown in red, and the nucleotide creating the intrinsic mismatch with the WT sequence at the SNV site is shown in blue.

**C, D.** Dual mismatch crRNAs (DM1-DM12) were designed by combining selected mismatch positions from M1-M23 series of *IDH1* crRNA1 (C) and *BRAF* crRNA2 (D). Additional engineered mismatches are shown in red, and the nucleotide creating the intrinsic mismatch with the WT sequence at the SNV site is shown in blue.

1. Structural model of the miniCas13d-crRNA-target RNA ternary complex predicted by AlphaFold3. M2-M6 represents the initial binding and nucleation window that mediates early target capture and association kinetics and is highly sensitive to mismatches. M17-M22 corresponds to the activation gating and nuclease switch window that regulates conformational rearrangement and HEPN catalytic activation, with mismatches primarily reducing cleavage efficiency rather than target binding.


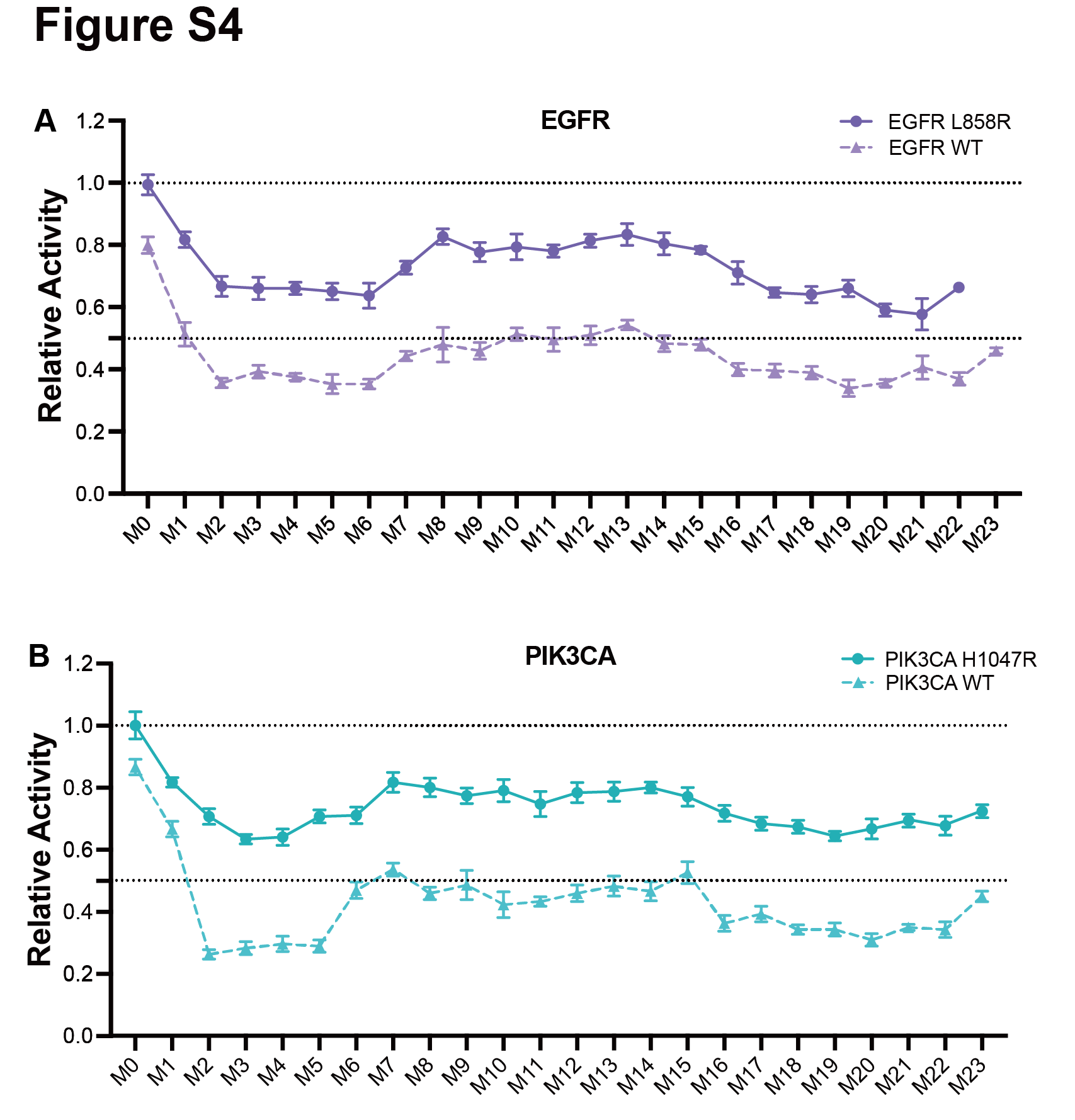


**Figure S4. Profiling of SNV discrimination using engineered mismatch crRNAs**

**A.** Relative cleavage activity of each mismatch variant was assessed against *EGFR* L858R (solid lines) and *EGFR* WT (dashed lines) RNA targets. Fluorescence signals were normalized to the activity of the reference crRNA (M0). Data are presented as mean ± SD (n = 3).

**B.** Relative cleavage activity of each mismatch variant was assessed against *PIK3CA* H1047R (solid lines) and *PIK3CA* WT (dashed lines) RNA targets. Fluorescence signals were normalized to the activity of the reference crRNA (M0). Data are presented as mean ± SD (n = 3).

**
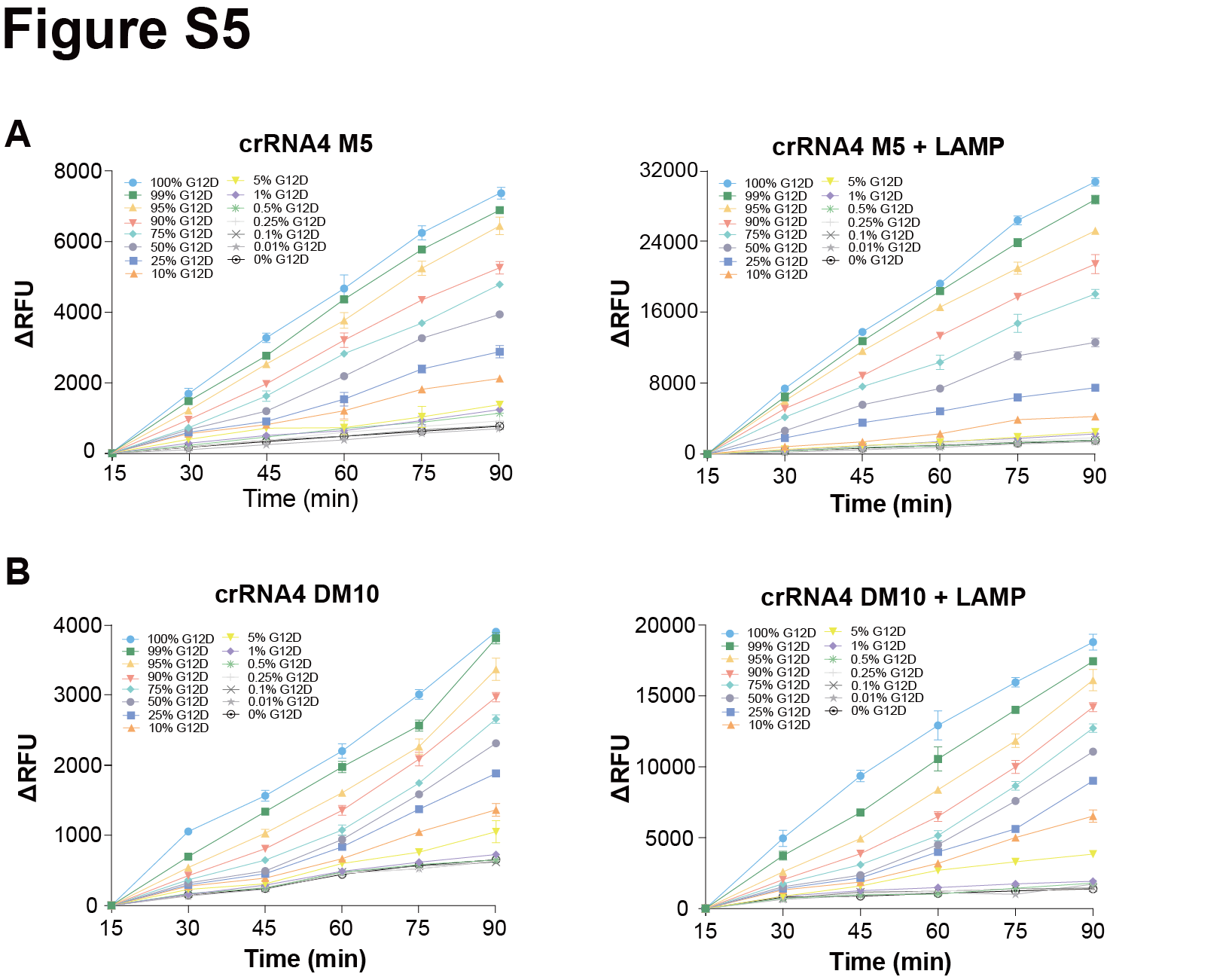
**

**Figure S5. Time course fluorescence analysis of miniCas13d based detection across *KRAS* G12D RNA dilutions**

1. Time-course fluorescence curves of miniCas13d guided by crRNA M5, across a dilution series of *KRAS* G12D RNA (100% to 0%), without and with LAMP amplification (left and right, respectively). ΔRFU was calculated as fluorescence intensity at each time point minus the baseline at 15 minutes. Data are presented as mean ± SD (n = 3).
   **B**. Time-course fluorescence curves of miniCas13d guided by crRNA DM10, across a dilution series of *KRAS* G12D RNA (100% to 0%), without and with LAMP amplification (left and right, respectively). ΔRFU was calculated as fluorescence intensity at each time point minus the baseline at 15 minutes. Data are presented as mean ± SD (n = 3).


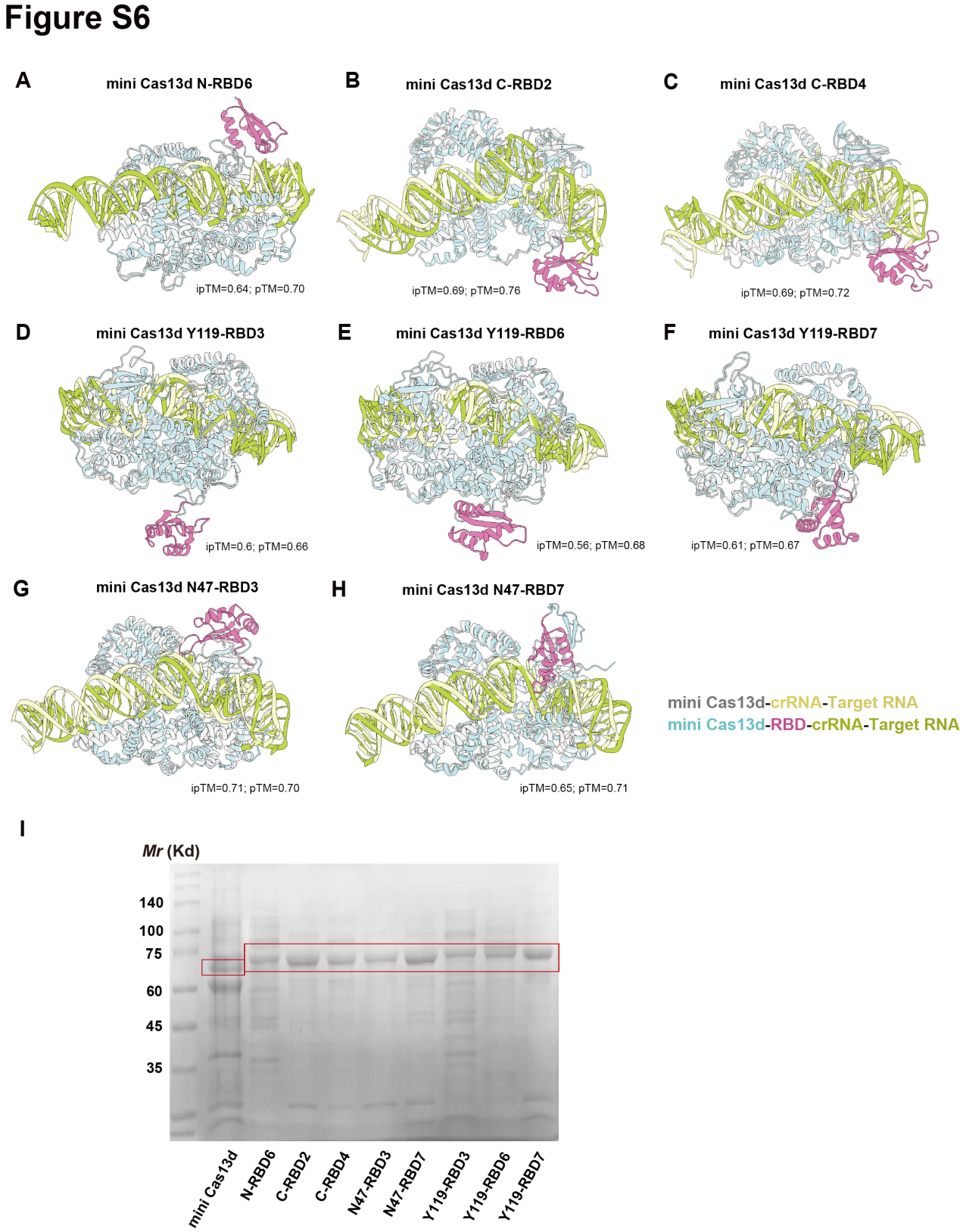


**Figure S6. Structural predictions of miniCas13d-RBD fusion proteins by AlphaFold3**

**A-H.** Engineered Cas13d variants with RNA binding domains (RBDs) inserted at indicated sites were structurally predicted using AlphaFold3. Predicted complexes are shown in cartoon, with the crRNA in green, miniCas13d in cyan, and inserted RBD in magenta. Each fusion model was structurally aligned to miniCas13d-crRNA-target RNA complex (shown in light grey) to assess potential conformational perturbations upon RBD insertion. Predicted inter chain template modeling scores (ipTM) and global predicted TM scores (pTM) are indicated.

**I.** SDS-PAGE analysis of purified miniCas13d-RBD variants (15-20 μg per lane). All variants exhibited a predominant ~75 kDa band (red box), consistent with the expected molecular weight after RBD insertion.

**
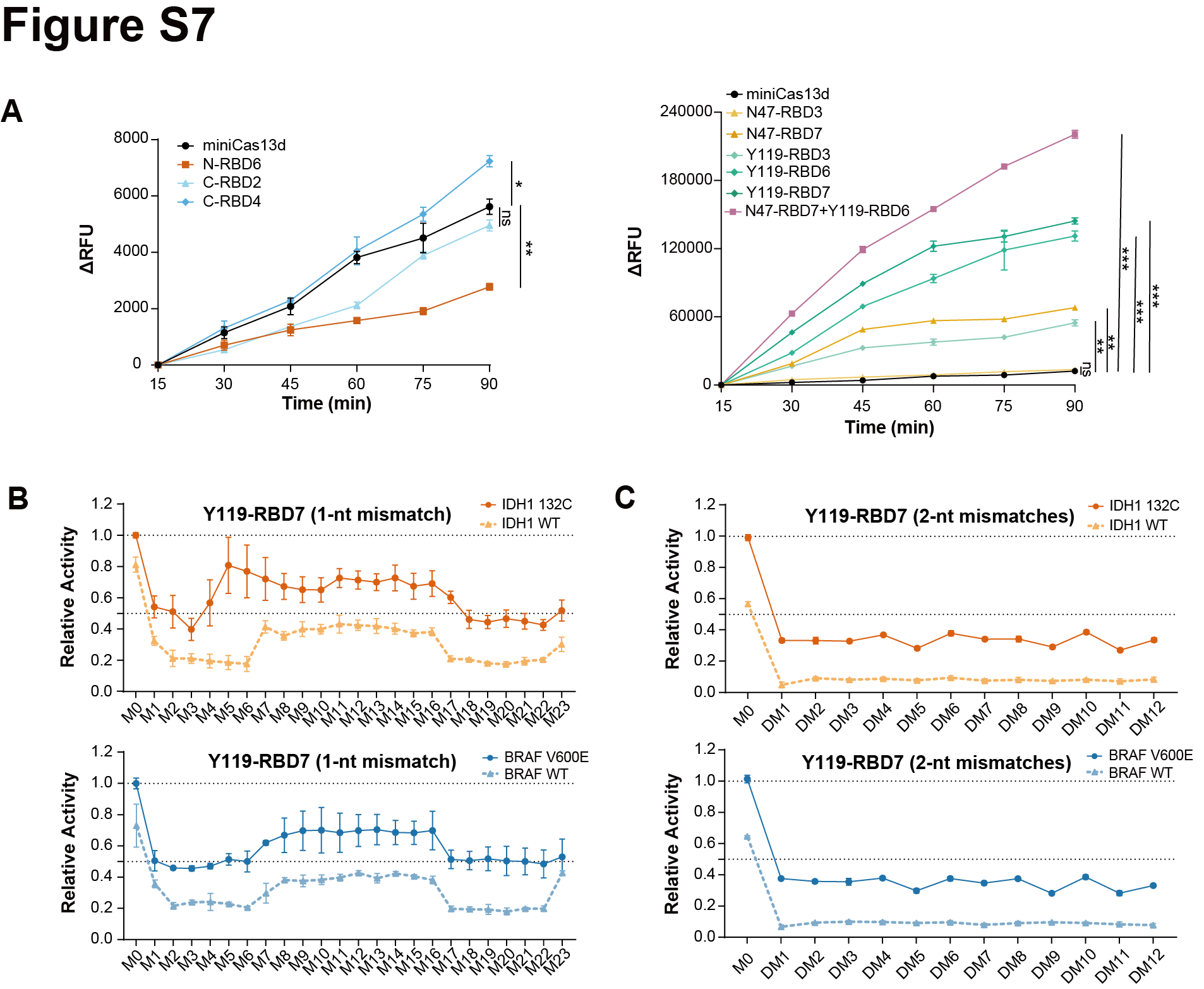
**

**Figure S7. Performance of engineered miniCas13d RBD variants for SNV detection across targets and mismatches**

**A.** Time course analysis of RNA cleavage activity by Cas13d variants targeting *KRAS* G12D RNA. Left panel: Terminal fusions of RBDs at the N-terminus (orange) and C-terminus (blue). Right panel: Internal RBD insertions at N47 (yellow), Y119 (green), and dual insertion at N47+Y119 (pink). Wild type miniCas13d is shown in black. Fluorescence values represent mean ± SD (n = 3). Statistical significance was determined using independent samples t-tests. *p < 0.05; **p < 0.01; ***p < 0.001; ns, not significant.

**B, C.** Mismatch discrimination profiling of Y119-RBD7 variant using crRNAs containing single nucleotide mismatches (B, M1-M23) or double mismatches (C, DM1-DM12). Relative cleavage activity against *IDH1* R132C (upper panels, orange) and *BRAF* V600E (lower panels, blue) was quantified and normalized to reference crRNA (M0). Solid and dashed lines represent SNV and WT RNA, respectively. Data represent mean ± SD (n = 3).


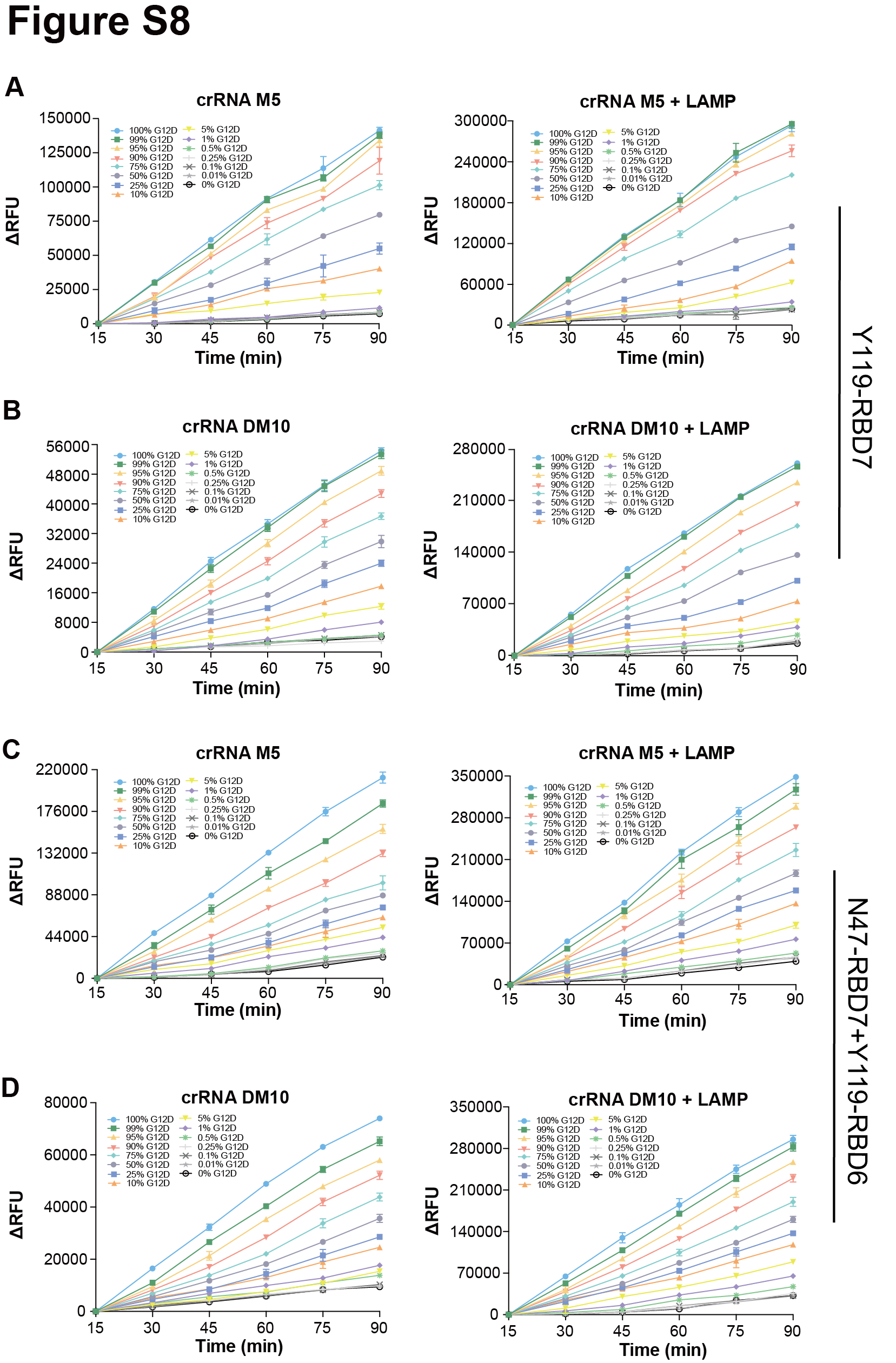


**Figure S8. Time course fluorescence analysis of engineered Cas13d variants across *KRAS* G12D RNA dilutions**

**A, B.** Time course fluorescence curves of Y119-RBD7 guided by crRNA M5 (A) and crRNA DM10 (B), across a dilution series of *KRAS* G12D RNA (100% to 0%), without (left) and with (right) LAMP amplification. ΔRFU was calculated as fluorescence intensity at each time point minus the baseline at 15 minutes. Data are presented as mean ± SD (n = 3).

**C, D.** Time course fluorescence curves of N47-RBD7+Y119-RBD6 guided by crRNA M5 (C) and crRNA DM10 (D), across a dilution series of *KRAS* G12D RNA (100% to 0%), without (left) and with (right) LAMP amplification. Data are presented as mean ± SD (n = 3).


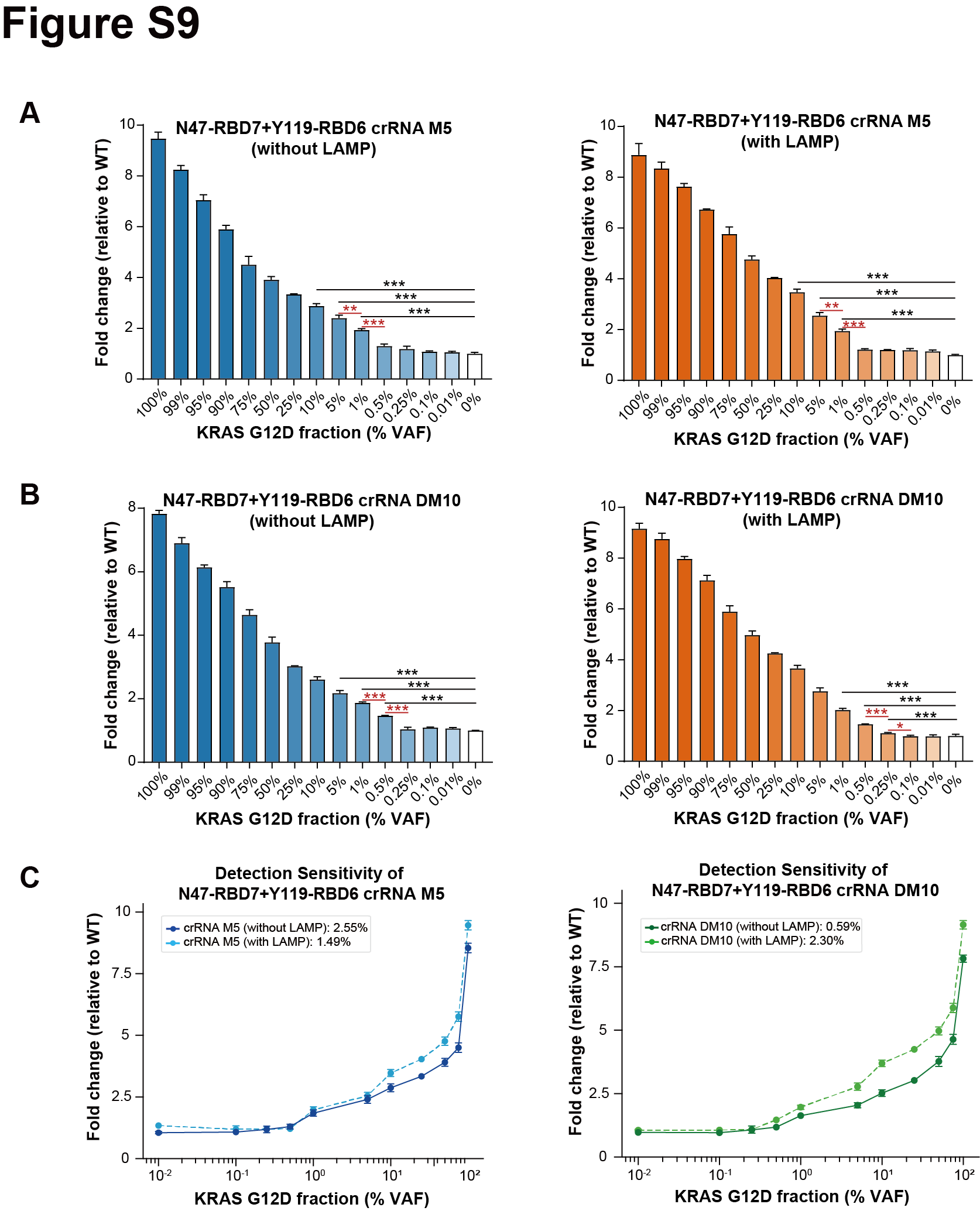


**Figure S9. Performance of engineered N47-RBD7+Y119-RBD6 for SNV detection**

**A, B.** Detection sensitivity analysis of N47-RBD7+Y119-RBD6 variant guided by mismatch optimized crRNAs M5 (A) and DM10 (B) across a *KRAS* G12D dilution series. Bar plots show fold changes in fluorescence relative to the WT control (0% G12D) without LAMP (blue) and with LAMP (orange). Data represent mean ± SD (n = 3). Statistical significance was assessed using independent samples t-tests. *p < 0.05; **p < 0.01; ***p < 0.001; ns, not significant.

**C.** Line plots showing fluorescence fold changes of N47-RBD7+Y119-RBD6 relative to WT across *KRAS* G12D RNA dilutions for crRNA M5 (left) and DM10 (right), measured without (solid lines) and with (dashed lines) LAMP. Data are presented as mean ± SD (n = 3).

**
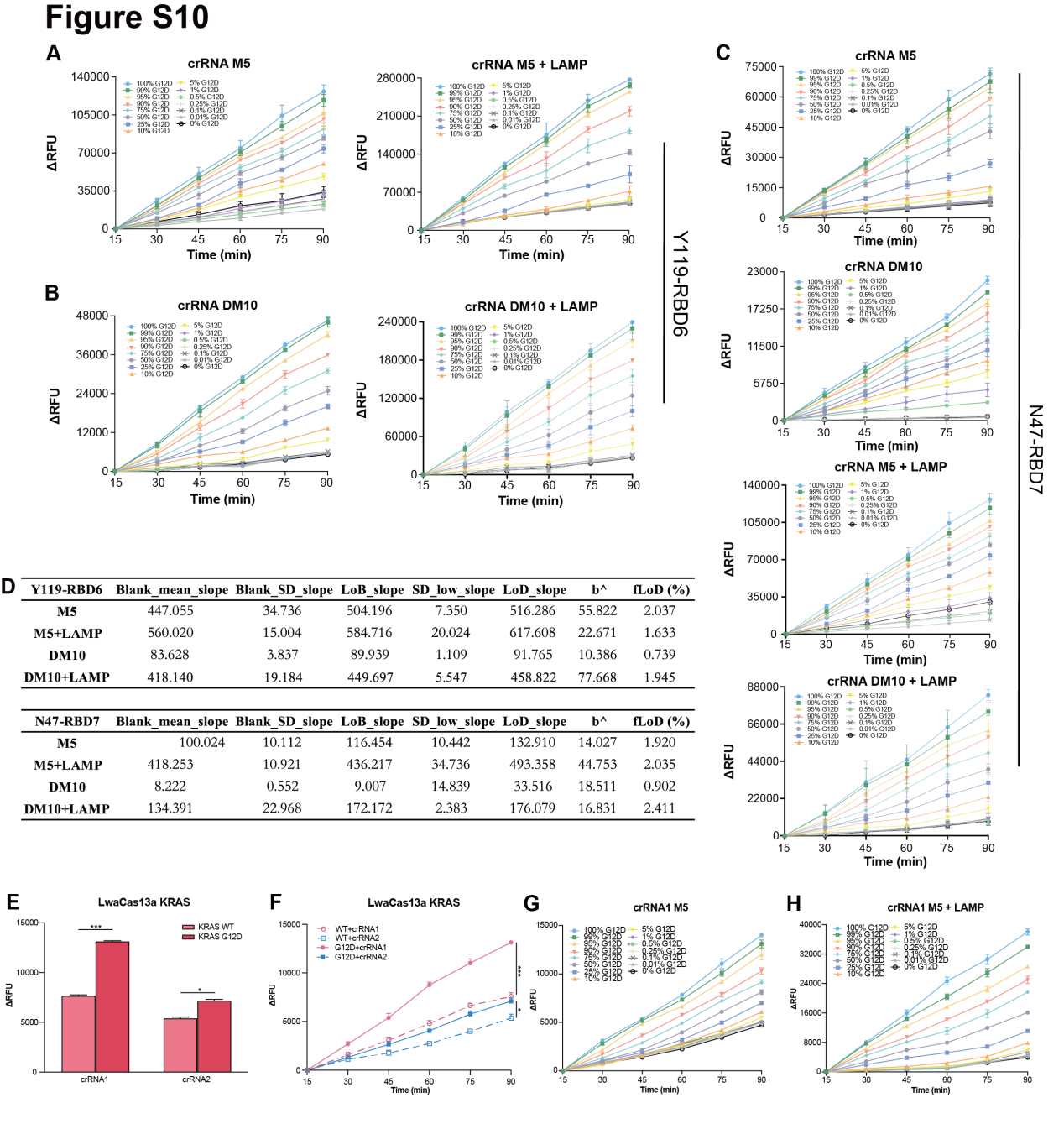
**

**Figure S10. Time course fluorescence analysis of engineered Cas13d variants and LwaCas13a across *KRAS* G12D RNA dilutions**

1. Kinetic fluorescence readouts for Y119‑RBD6 variant using crRNA M5 across a *KRAS* G12D variant allele fraction dilution series, without amplification (left) and with LAMP amplification (right).
2. Kinetic fluorescence readouts for Y119‑RBD6 variant using the dual-mismatch guide DM10 across the same *KRAS* G12D dilution series, without amplification (left) and with LAMP amplification (right).
3. Corresponding kinetic fluorescence readouts for the N47-RBD7 variant using crRNA M5 and DM10, each measured without amplification and with LAMP amplification, as shown in four subpanels.
4. CLSI EP17‑A2 analytical performance summary for Y119‑RBD6 and N47‑RBD7 across the indicated guide and amplification configurations, reporting Blank_mean_slope, Blank_SD_slope, LoB_slope, SD_low_slope, LoD_slope, fitted slope term (b̂), and functional LoD (fLoD, %VAF).
5. Benchmarking of LwaCas13a for discrimination between *KRAS* WT and *KRAS* G12D using two crRNAs. Statistical significance is indicated. *p < 0.05; ***p < 0.001.
6. Representative kinetic traces for the LwaCas13a benchmarking reactions shown in panel E, including WT or G12D targets paired with crRNA1 or crRNA2.
7. LwaCas13a dilution‑series kinetics using the mismatch guide crRNA1 M5 across *KRAS* G12D variant allele fractions without amplification.
8. LwaCas13a dilution-series kinetics using the mismatch guide crRNA1 M5 across *KRAS* G12D variant allele fractions with LAMP amplification.

All fluorescence traces are shown as mean ± SD (n = 3 independent replicates).


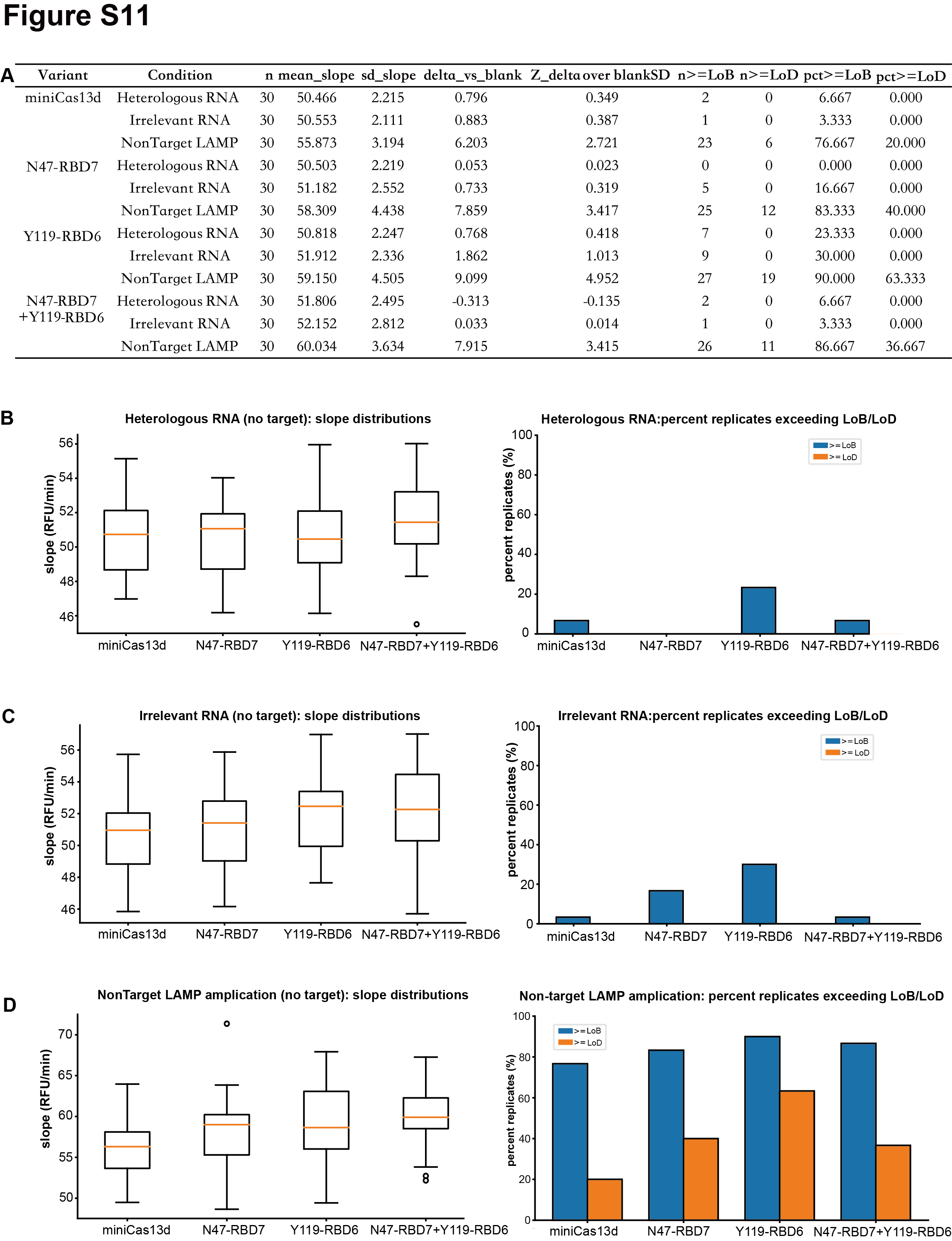


**Figure S11. Background activation profiling across Cas13d scaffold variants under target-absent conditions**

1. **Summary table of initial slope statistics for collateral reporter cleavage measured under heterologous RNA, irrelevant RNA, and non-target LAMP background conditions across Cas13d variants. Reported metrics include replicate number (n), mean slope, slope SD, mean shift relative to the blank (Δ vs blank), Z score calculated relative to the blank SD, and the number and percentage of reactions exceeding the EP17-derived limit of blank (LoB) and limit of detection (LoD).**
2. **Heterologous RNA background. Left, boxplots of slope distributions for each Cas13d variant. Right, percentages of replicates exceeding LoB and LoD.**
3. **Irrelevant RNA background. Left, boxplots of slope distributions for each Cas13d variant. Right, percentages of replicates exceeding LoB and LoD.**
4. **Non‑target LAMP background. Left, boxplots of slope distributions for each Cas13d variant. Right, percentages of replicates exceeding LoB and LoD.**


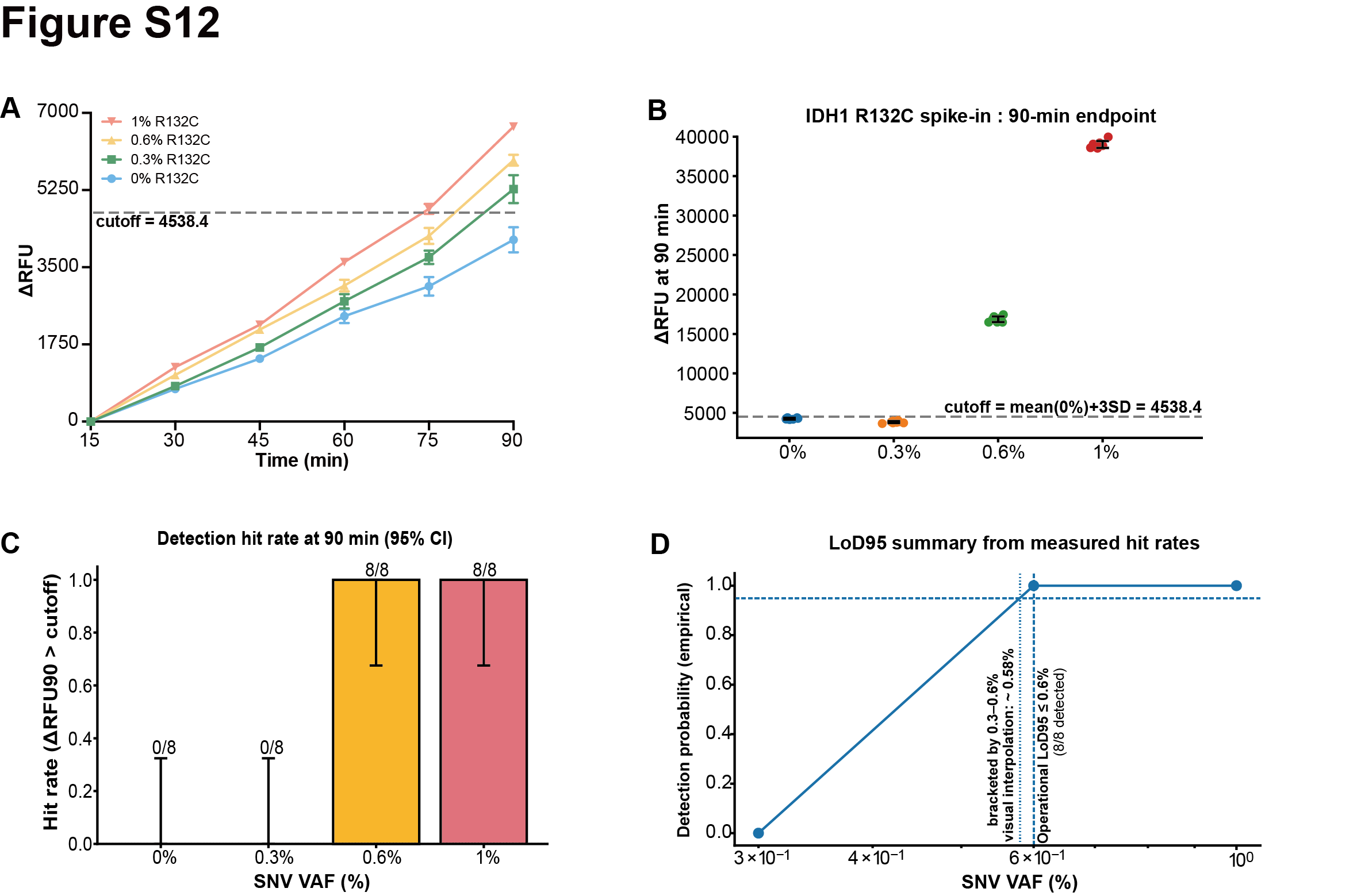


**Figure S12. Amplification-free spike-in validation in a clinical RNA matrix for *IDH1* R132C at sub-1% VAF**

1. **Time‑course fluorescence trajectories for *IDH1* R132C spike‑in mixtures prepared by adding in vitro-transcribed mutant RNA to RNA extracted from an *IDH1* R132C negative clinical specimen. Samples contained 0%, 0.3%, 0.6%, or 1% mutant allele fraction, with 8 replicates per level. Signals are plotted as ΔRFU relative to the 15‑min baseline. Error bars indicate mean ± SD. The dashed line denotes the prespecified decision cutoff derived from 0% controls.**
2. **90‑min endpoint readout for the same spike‑in series. Points represent individual replicates, and error bars indicate mean ± SD. The dashed line indicates the fixed cutoff calculated as mean(0%) + 3×SD.**
3. **Empirical detection hit rate at 90 min, defined as the fraction of replicates exceeding the cutoff, with 95% binomial confidence intervals. Numbers above the bars indicate positives over total replicates.**

**D. LoD_95_ estimation from measured hit rates. Detection probability is plotted versus input VAF, and LoD_95_ is estimated by interpolation at 95% detection probability (dashed guides).**
